# Polymorphisms in the Estrogen Receptor 1 and Vitamin C and Matrix Metalloproteinase Gene Families Are Associated with Susceptibility to Lymphoma

**DOI:** 10.1371/journal.pone.0002816

**Published:** 2008-07-30

**Authors:** Christine F. Skibola, Paige M. Bracci, Eran Halperin, Alexandra Nieters, Alan Hubbard, Randi A. Paynter, Danica R. Skibola, Luz Agana, Nikolaus Becker, Patrick Tressler, Matthew S. Forrest, Sriram Sankararaman, Lucia Conde, Elizabeth A. Holly, Martyn T. Smith

**Affiliations:** 1 School of Public Health, Division of Environmental Health Sciences, University of California Berkeley, Berkeley, California, United States of America; 2 Department of Epidemiology and Biostatistics, University of California San Francisco, San Francisco, California, United States of America; 3 International Computer Science Institute, Berkeley, California, United States of America; 4 Division of Cancer Epidemiology, German Cancer Research Center, Heidelberg, Germany; 5 Department of Pathology, University of California San Francisco, San Francisco, California, United States of America; 6 Wellcome Trust Sanger Institute, Wellcome Trust Genome Campus, Hinxton, United Kingdom; New York University School of Medicine, United States of America

## Abstract

**Background:**

Non-Hodgkin lymphoma (NHL) is the fifth most common cancer in the U.S. and few causes have been identified. Genetic association studies may help identify environmental risk factors and enhance our understanding of disease mechanisms.

**Methodology/Principal Findings:**

768 coding and haplotype tagging SNPs in 146 genes were examined using Illumina GoldenGate technology in a large population-based case-control study of NHL in the San Francisco Bay Area (1,292 cases 1,375 controls are included here). Statistical analyses were restricted to HIV- participants of white non-Hispanic origin. Genes involved in steroidogenesis, immune function, cell signaling, sunlight exposure, xenobiotic metabolism/oxidative stress, energy balance, and uptake and metabolism of cholesterol, folate and vitamin C were investigated. Sixteen SNPs in eight pathways and nine haplotypes were associated with NHL after correction for multiple testing at the adjusted *q*<0.10 level. Eight SNPs were tested in an independent case-control study of lymphoma in Germany (494 NHL cases and 494 matched controls). Novel associations with common variants in estrogen receptor 1 (*ESR1*) and in the vitamin C receptor and matrix metalloproteinase gene families were observed. Four *ESR1* SNPs were associated with follicular lymphoma (FL) in the U.S. study, with rs3020314 remaining associated with reduced risk of FL after multiple testing adjustments [odds ratio (OR) = 0.42, 95% confidence interval (CI) = 0.23–0.77) and replication in the German study (OR = 0.24, 95% CI = 0.06–0.94). Several SNPs and haplotypes in the matrix metalloproteinase-3 (*MMP3*) and *MMP9* genes and in the vitamin C receptor genes, solute carrier family 23 member 1 (*SLC23A1*) and *SLC23A2*, showed associations with NHL risk.

**Conclusions/Significance:**

Our findings suggest a role for estrogen, vitamin C and matrix metalloproteinases in the pathogenesis of NHL that will require further validation.

## Introduction

Non-Hodgkin lymphoma (NHL) is the most common hematopoietic cancer diagnosed in the U.S. Rates have increased steadily over the last 30 years (http://www.leukemia-lymphoma.org). Known risk factors include family history, exposure to infectious agents and immunodeficiency associated with congenital conditions and post-transplant therapy[1], though the causes for most cases are unknown. Genetic and environmental factors that influence the growth, survival and capacity of B-cells with pre-neoplastic changes to undergo transformation[2] or that impact the quality of tumor-specific immune responses may play integral roles in lymphomagenesis. Approximately 80% of lymphomas are of B-cell origin that arise during germinal center (GC) and post-GC reactions as part of otherwise normal B-cell differentiation[3]. Diffuse large B-cell lymphoma (DLBCL), follicular lymphoma (FL) and small lymphocytic lymphoma/chronic lymphocytic leukemia (SLL/CLL) are major lymphoma histologies that collectively comprise ∼75% of all NHL in industrialized countries. Whereas DLBCL is a clinically aggressive disease for which incidence increases with age, FL and SLL/CLL present a more indolent clinical course. Molecular and phenotypic differences between NHL histologies suggest they may have distinct etiologies, though overlapping mechanisms are likely. The study of candidate single nucleotide polymorphisms (SNPs) may help to identify environmental risk factors, further elucidate disease mechanisms and discover novel targets for lymphoma treatment regimens. Thus, we undertook a genetic study of 768 coding and haplotype tagging SNPs in 146 genes in a population-based case-control study of NHL in the San Francisco Bay Area (1,292 cases, 1,375 controls). The genes investigated were primarily selected based on findings from our earlier genetic studies[2], [4]–[12] in pathways involved in steroidogenesis, immune function, cell signaling, sunlight exposure, xenobiotic metabolism/oxidative stress, energy balance, as well as uptake and metabolism of cholesterol, folate and vitamin C (Table 1). The most compelling associations were tested in an independent population-based case-control study of lymphoma based in Germany.

**Table 1:** Pathways/gene functional groups of analyzed candidate genes in the San Francisco Bay Area NHL study.

Pathway/Gene Function	
Steroidogenesis (n = 22)	*CYP11B1, CYP11B2, CYP21A2, NR3C1, AR, CYP17A1, CYP19A1, COMT, ESR1, ESR2, HSD17B1, HSD17B2, HSD3B1, HSD3B2, NCOA3, PGR, PRL, PRLR, SERPINA6, SHBG, SRD5A1, SRD5A1*
Immunity (n = 22)	*IL10, 1L4, IL4R, CTLA4, FCER2, FCGR2A, FCGR3A, VCAM1, ICAM1, C4A, HLA-DQB1, NFKB1, NFKBIA, NFKBIB, TNFRSF5, TNFSF8, CCL2, CCL5, CCL8, CCR3, CX3CR1, CXCR4*
Cell signaling/apoptosis (19)	*BAD, BAX, BAK1, BCL2, BCL2L1, BCL6, BIK, FLT1, ITGB2, NFKB1, NFKB1A, MMP1, MMP2, MMP3, MMP9, VEGF, SOCS3, STAT3, STAT6*
Sunlight/vitamin D (n = 12)	*CDK4, CDKN2A, CDKN2B, ERCC2, MC1R, MITF, OCA2, TYR, VDR, VDRIP, XRCC1, POMC*
Xenobiotic metabolism/oxidative stress (n = 14)	*AHR, CYP1A1, CYP2E1, EPHX1, EPHX2, NAT1, NAT2, PTGS1, PTGS2, GPX4, MPO, SOD1, SOD2, NQO1*
Energy balance (n = 38)	*ADRB2, ADRB3, APOA2, APOA4, APOB, APOE, ESR1, GH1, GHR, GHRL, HP, IGFI, IGFIR, IGF2, IGF2R, IGFBP1, IGFBP3, LEP, LEPR, LTA, NPY, POMC, PARP1, PPARD, PPARG, PTPN1, SIRT1, TNFRSF1B, CYP11B1, CYP21A2, NR3C1, DRD2, DRD3, DRD5, SLC6A3, COMT, PRL, PRLR*
Cholesterol synthesis/metabolism (n = 19)	*ACBD3, ADAM17, BZRP, CETP, FDFT1, HMGCS1, HMGCS2, LIPC, LPL, NPC1, NPC2, NR1H2, NR1H3, STAR, STARD3NL, TIMP1, TIMP3, TNFSF8, TPRT*
Diet	
Folate metabolism (n = 7)	*ADH5, CBS, DHFR, MTHFR, MTR, SHMT2, TYMS*
Vitamin C uptake (n = 2)	*SLC23A1, SLC23A2*

## Results

### Assessment of sample completion rates and SNP call rates

Of the 768 SNPs genotyped in DNA of 2,667 study participants (1,292 cases and 1,375 controls) from a large population-based case-control study with more than 4,100 participants, eight samples were excluded from further analyses due to missing SNP data (>10%) yielding a sample completion rate of 99.7%. After restriction to white non-Hispanics, 959 cases and 1,049 controls were included in the final analysis. A total of 62 SNPs with call rates <90% were dropped from the analysis yielding a SNP call rate of 91.9%. Additional dropped SNPs included those with minor allele frequencies <5% (n = 29), X chromosome SNPs (n = 7) and SNPs with Hardy-Weinberg equilibrium *p*-values<0.01 (n = 33) leaving a total of 637 SNPs for subsequent analyses. Two pairs of replicate DNAs on each plate revealed genotype concordance rates of >99% within and between plates. Genotype results concurred (>98.5%) with data from the quality control Taqman genotyping of 10 SNPs on the same study population. Single-marker allelic tests for all SNPs were performed using χ2 statistics. SNPs and haplotypes with the strongest statistical associations (*q*-values≤0.10) adjusted for multiple comparisons using the false discovery rate (FDR) are shown in Tables 2 and 3, respectively, while *p*-values for all SNPs are available online (website). None of the associated SNPs violated HWE at the *p*<0.05 level (Table 2).

**Table 2:** SNPs associated with NHL with corrected *p*-value*s* (*q*)<0.10* for the San Francisco Bay Area NHL case-control study.

Pathway	Chromosome location	dbSNP ID	Location	Gene Symbol	Gene Name	HWE *p*-value	Raw *p*-value *	FDR (BH) *q*-value	Control MAF	OR (95% CI) Var/var vs. Wt/wt
**All NHL**										
Diet, Vitamin C	chr20:4879113	rs1776948	IVS2-17750G>A	*SLC23A2*	Solute carrier family 23 (nucleobase transporters), member 2	.89	**4.7×10^−3^**	**4.3×10^−2^**	.44	**1.4 (1.1–1.9)**
**FL**										
Steroidogenesis	chr15:49412515	rs1870049	IVS1+5469T>C	*CYP19A1*	Cytochrome P450, family 19, subfamily A, polypeptide 1	.78	**6.0×10^−4^**	**4.9×10^−2^**	.12	**3.0 (1.2–7.5)**
	chr6:152362786	rs3020314	IVS4+5029T>C	*ESR1*	Estrogen receptor 1	.96	**1.6×10^−3^**	7.0×10^−2^	.34	**0.42 (.23–.77)**
Cell signaling	chr11:102218830	rs679620	45K>E (657A>G)	*MMP3*	Matrix metallopeptidase 3	.35	**3.7×10^−4^**	**1.2×10^−2^**	.50	**1.7 (1.1–2.6)**
	chr21:45152527	rs760462	IVS3+2100G>A	*ITGB2*	Integrin, beta 2	.46	9.7×10^−4^	7.2×10^−2^	.21	3.1 (1.3–6.9)
Cholesterol	chr8:11697771	rs1047643	7L>L (20T>C)	*FDFT1*	Farnesyl-diphosphate farnesyltransferase 1	.22	8.4×10^−4^	5.2×10^−2^	.35	1.9 (1.3–2.8) var
Diet, vitamin C	chr5:138742373	rs11950646	IVS9-110A>G	*SLC23A1*	Solute carrier family 23 (nucleobase transporters), member 1	.42	2.7×10^−2^	8.4×10^−2^	.35	1.8 (1.2–2.9)
	chr5:138738475	rs6596473	IVS13+2515G>C			.36	2.5×10^−2^	8.4×10^−2^	.32	1.8 (1.2–2.8)
	chr20:4879113	rs1776948	IVS2-17750G>A	*SLC23A2*	Solute carrier family 23 (nucleobase transporters), member 2	.89	2.0×10^−2^	8.4×10^−2^	.44	1.6 (1.1–2.4)
**SLL/CLL**										
Diet, vitamin C	chr20:4839759	rs6133175	IVS4+1767A>G	*SLC23A2*	Solute carrier family 23 (nucleobase transporters), member 2	.91	**1.9×10^−2^**	**4.4×10^−2^**	.33	**1.9 (1.1–3.2)**
	chr20:4846896	rs1715364	IVS3+14205T>C			.38	**3.3×10^−3^**	**3.0×10^−2^**	.44	**1.9 (1.2–3.1)**
	chr20:4879113	rs1776948	IVS2-17750G>A			.89	**1.0×10^−3^**	**3.0×10^−2^**	.44	**2.0 (1.2–3.2)**
Cell signaling	chr20:44073632	rs17576	279Q>R (2659A>G)	*MMP9*	Matrix metallopeptidase 9	.72	7.0×10^−3^	9.5×10^−2^	.36	1.8 (1.1–3.0)
	chr20:44067957	rs3918241	−1831T>A promoter			.83	**1.0×10^−2^**	9.5×10^−2^	.15	**1.6 (1.1–2.3) var**
		rs2274756	668R>Q			.74	1.0×10^−2^	9.5×10^−2^	.14	1.6 (1.1–2.3) var
Xenobiotic metabolism	chr8:27433079	rs4149244	IVS9+1573C>T	*EPHX2*	Top of Form Epoxide hydrolase 2 Bottom of Form	.63	**1.2×10^−3^**	**4.5×10^−2^**	.10	**2.0 (1.2–3.4)**

MAF: minor allele frequency; NHL: non-Hodgkin lymphoma; FL: follicular lymphoma; DLBCL: diffuse large B-cell lymphoma; SLL: small lymphocytic lymphoma; CLL: chronic lymphocytic leukemia.

* *q*<0.05 are in bold.

**Table 3:** Associated haplotypes with corrected *p*-value*s* (*q*)<0.10 for the San Francisco Bay Area NHL case-control study.

Subtype	Pathway	Gene	rs id	Haplotype	*p*-value	*q*-value	OR (95% CI)
All NHL	Vitamin C	*SLC23A2*	rs1776948, rs6139587	10 (AA)	**2.0×10^−3^**	**4.6×10^−2^**	**1.2 (1.02–1.4)**
			rs1776948, rs6139587	01 (GT)	**2.0×10^−3^**	**4.6×10^−2^**	**0.82 (.69–.97)**
			rs1715385, rs6133175, rs1715364	101 (AAC)	**8.0×10^−4^**	**4.6×10^−2^**	**1.4 (1.07–1.8)**
FL	Cholesterol	*FDFT1*	rs2645444, rs1047643, rs3258, rs1296028	0100 [28]	1.6×10^−3^	6.4×10^−2^	1.7 (1.2–2.3)
	Steroidogenesis	*CYP19A1*	rs1004982, rs2470146, rs2470144, rs1870049, rs752760	10011 (GTGCT)	**6.5×10^−4^**	**2.9×10^−2^**	**1.7 (1.3–2.4)**
DLBCL	Vitamin C	*SLC23A2*	rs1715385, rs6133175, rs1715364	101 (AAC)	**7.3×10^−3^**	**4.4×10^−2^**	**1.6 (1.1–2.4)**
SLL/CLL	Vitamin C	*SLC23A2*	rs1776948, rs6139587	10 (AA)	**1.8×10^−3^**	**1.1×10^−2^**	**1.7 (1.2–2.3)**
	Cell signaling/apoptosis	*BIK*	rs747745, rs9620115, rs5751435, rs11574528, rs13815	01011 (AGCTC)	**8.0×10^−4^**	**3.7×10^−2^**	**2.1 (1.4–3.4)**
	Xenobiotic/oxdative stress	*EPHX2*	rs4149244, rs747276	11(TC)	**1.0×10^−3^**	**2.0×10^−2^**	**2.1 (1.3–3.3)**

NHL: non-Hodgkin lymphoma; FL: follicular lymphoma; DLBCL: diffuse large B-cell lymphoma; SLL: small lymphocytic lymphoma; CLL: chronic lymphocytic leukemia.

### Gene- and SNP-based analyses

#### SNPs in vitamin C receptor genes influence risk of NHL, FL and SLL/CLL

SNP-specific analyses revealed that both SNPs genotyped in the vitamin C receptor gene, solute carrier family 23 member 1 (*SLC23A1*), rs6596473 (IVS13+2515G>C) and rs11950646 (IVS9−110A>G), influenced risk of FL (*p* = 2.5×10^−2^, *q* = 8.4×10^−2^, Table 2). Those homozygous variant for either SNP had an 80% elevated risk of FL (Table S1). These SNPs captured 75% of the haplotype structure in *SLC23A1* (R^2^ = 0.99) though no haplotype associations were found.

An intronic *SLC23A2* SNP rs1776948 (IVS2−17750G>A) influenced NHL (*p* = 4.7×10^−3^, *q* = 4.3×10^−2^), FL (*p* = 2.0×10^−2^, *q* = 8.4×10^−2^) and SLL/CLL risk (*p* = 1.1×10^−3^, *q* = 3.0×10^−2^). In logistic regression analyses, the rs1776948 homozygous variant 17750AA genotype was positively associated with disease risk (Table S1). Notable associations were observed for two additional intronic *SLC23A2* SNPs, rs6133175 (IVS4+1767A>G) and rs1715364 (IVS3+14205T>C) (*q*<0.05), where the homozygous variant genotypes yielded two-fold increased risks for SLL/CLL (Table S1). TFSEARCH (http://www.cbrc.jp/research/db/TFSEARCH.html) predicted that the *SLC23A2* rs1715364 wild type T allele creates binding sites for the GATA-1 and GATA-2 enhancer proteins that may increase *SLC23A2* expression. LD structure for *SLC23A2* is shown in Figure S1. Given the size of this gene (∼158 Kb), the seven *SLC23A2* tag SNPs genotyped only captured 35% of its haplotype structure at the R^2^ = 0.94 level. Though gene coverage was low, haplotype associations were found with NHL, DLBCL and SLL/CLL risk (Table 3). A two-SNP AA haplotype (rs1776948 and rs6139587) was positively associated with NHL and SLL/CLL and an AAC haplotype derived from minor alleles for rs1715385 and rs1715364 and the wildtype allele for rs6133175 was associated with increased risk of NHL and DLBCL. However, *p*-values derived from *SLC23A2* haplotype associations predicted no greater risks beyond those observed for the individual SNPs.

#### Matrix metalloproteinase (MMP)3 and MMP9 SNPs influence FL and SLL/CLL risk

A significant association was found between FL and a non-synonymous *MMP3* 45K>E SNP, (rs679620, A>G) (*p* = 3.7×10^−4^; *q* = 1.2×10^−2^; Table 2). Logistic regression analyses revealed that the *MMP3* 45EE allelotype was associated with a 70% increased risk for FL (Table S2). RESCUE ESE[13] predicted that the *MMP3* 45E variant introduces two exonic splicing enhancer motifs that could augment exon 2 splicing and increase *MMP3* expression. This SNP, together with rs615098, located in the *MMP3* promoter region, captured 44% of all common HapMap SNPs in the region at a mean R^2^ of 0.99.

Two non-synonymous SNPs in *MMP9* (279Q>R, rs17576; 668R>Q, rs2274756) influenced SLL/CLL status (*p* = 7.0×10^−3^, *q* = 9.5×10^−2^; *p* = 1.0×10^−2^, *q* = 9.5×10^−2^, respectively; Tables 2, S2). For the 668Q allele, ESEfinder identified an extra SF2/ASF motif that could enhance exon 2 splicing and increase *MMP9* expression. Interestingly, the 668R>Q SNP was in complete LD with a *MMP9* promoter SNP, rs3918241 (D' = 1.0), where the variant A allele was positively associated with SLL/CLL (*p* = 1.0×10^−2^; *q* = 9.5×10^−2^; Tables 2, Table S2). TFSEARCH predicts that the rs3918241 A allele creates extra GATA-1, Oct-1, GATA-2 and GATA-X binding motifs, suggesting increased promoter activity for the variant A allele. *MMP9* variation was organized into a single LD block spanning the gene and its promoter region (Figure S2). Thus, the five *MMP9* SNPs genotyped captured 78% of all common variation for this region at a mean R^2^ of 0.98.

#### Sex hormone SNPs influence risk of lymphoma

The regression analyses (see methods) revealed an association between the cytochrome P450 (*CYP*)*19A1* gene and risk for FL (*p* = 6.3×10^−3^). Eight *CYP19A1* SNPs influenced risk for FL and SLL/CLL, though considering a *q*<0.10, only rs1870049 (IVS1+5469T>C) remained associated with disease status (*p* = 6.0×10^−4^; *q* = 4.9×10^−2^, Table 2). In logistic regression analyses, the rs1870049 homozygous variant CC genotype was associated with a three-fold increased risk for FL (Table S3). Four additional *CYP19A1* SNPs, rs10046 (3′UTR, 1678T>C), rs2289105 (IVS7−79T>C), rs2899472 (IVS4−1334C>A), rs9944225 (IVS3+7029C>A) and rs4774584 (IVS1−26860G>A) influenced SLL/CLL risk (Table S2) and rs2008691 (IVS1−13201A>G) was positively associated with the group of “other” lymphoma subtypes (Table S3). *CYP19A1* SNP associations did not differ by sex (data not shown). We also found a *CYP19A1* five-SNP high-risk FL haplotype (*p* = 6.5×10^−4^, *q* = 2.9×10^−2^, Table 3) that included the rs1870049 variant C allele. A haplotype with the rs1870049 T allele did not influence FL risk, suggesting that the C allele was driving the haplotype association. Analysis of LD structure throughout the region revealed that the haplotype lies within an 18 kb region of relatively high LD spanning the 5′ UTR of *CYP19A1* (Figure S3). The 14 genotyped SNPs, uniformly distributed throughout the gene, captured 66% of *CYP19A1* common genetic variation at a mean R^2^ of 0.96.

An intronic SNP, rs3020314 (IVS4+5029T>C), in the estrogen receptor alpha (*ESR1*) gene was associated with risk of FL (*p* = 1.6×10^−3^, *q* = 7.0×10^−2^, Table 2). Logistic regression analyses revealed that the rs3020314 homozygous variant IVS4+5029CC genotype was associated with a 58% reduced risk of FL (Table S4). This SNP was in almost complete LD (D'>0.9,R^2^>0.90) with three other SNPs that form part of a 14 kb haplotype block of 9 common SNPs located between exons 4 and 5. Homozygous variant genotypes for three additional *ESR1* SNPs, rs488133 (promoter−3604C>T), rs532010 (IVS1+1419T>C) and rs827423 (IVS1−7534T>C) also were associated with FL (Table S4). These three SNPs formed part of a major haplotype block that extended 43 kb from the 5′ upstream region to exon 2, which includes another 32 HapMap genotyped SNPs. LD structure analysis (Figure S4) revealed that some of the 11 *ESR1* SNPs genotyped in this study are located at the borderlines of blocks close to recombination hotspots. These SNPs, at a density of one every ∼28 Kb, captured only 25% of the common variation in *ESR1*.

#### Other genetic associations with NHL

In the cholesterol genes, considering *q*-values<0.10, we found an association between NHL and the nuclear receptor subfamily 1, group H, member 2 (*NR1H2*) gene (*p* = 1.8×10^−3^, *q* = 3.5×10^−2^) in the regression analysis. *NR1H2* is involved in cholesterol homeostasis and bile acid metabolism. For FL, we found gene and SNP (*20T>C*) associations with farnesyl-diphosphate farnesyltransferase 1(*FDFT1*), which plays a key role in cholesterol biosynthesis (gene, *p* = 4.1×10^−3^; *q* = 7.8×10^−2^; rs1047643, *p* = 8.4×10^−4^, *q* = 5.2×10^−2^, Table 2). According to ESEfinder, the rs1047643 C allele introduces an extra SRp55 motif that may influence pre-mRNA processing or splicing efficiency. This SNP was included in a FL high-risk *FDFT1* haplotype (*p* = 1.6×10^−3^, *q* = 6.4×10^−2^, Table 3), though the *p*-value was no more significant for the haplotype than for the rs1047643 SNP association. No other *FDFT1* SNP associations were observed. The four *FDFT1* SNPs genotyped captured 52% of common variation in *FDFT1* (R^2^ = 0.92).

In cell signaling genes, we found an association with NHL and the *BCL6* gene (*p* = 3.4×10^−3^, *q* = 7.8×10^−2^), though no single SNP significantly influenced risk. We also found that an intronic SNP rs760462 (IVS3+2100G>A) in integrin beta 2 (*ITGB2*) influenced FL status (*p* = 9.7×10^−4^, *q* = 7.2×10^−2^, Table 2), where the 2100AA genotype increased FL risk three-fold (Table 3). No other associations were found with *ITGB2*, however the six SNPs genotyped captured only 21% of the variation in the *ITGB2* gene (R^2^ = 0.99).

For SLL/CLL, the variant allele for rs4149244 (IVS9+1573C>T) in the epoxide hydrolase 2 (*EPHX2*) gene was positively associated with disease risk (*p* = 1.2×10^−3^, *q* = 4.5×10^−2^, Table 2). TFSEARCH predicts that the T allele introduces a CdxA binding site, suggesting that this allele may act to enhance *EPHX2* gene expression. Gene coverage for the four *EPHX2* SNPs genotyped was 58% (R^2^ = 0.96). The rs414924 T allele also comprised a two-SNP haplotype that was associated with FL risk (*p* = 1.0×10^−3^, *q* = 2.0×10^−2^, Table 3), though again the haplotype was no more likely to contribute to risk than the rs4149244 SNP. Finally, a five-SNP haplotype in the apoptosis/signaling gene, BCL2-interacting killer (*BIK*) was associated with an increased risk for SLL/CLL (Table 2), though no SNP associations were detected. Gene coverage for the seven *BIK* SNPs genotyped was 75% (R^2^ = 0.95).

### Validation Study

We next tested for association of significant findings in an independent case-control study of lymphoma in Germany that comprised 494 NHL cases and 494 age and sex-matched controls. An additional 111 Hodgkin lymphoma cases and 111 matched controls also were analyzed. The criterion used for selection of SNPs in Table 2 was that there was >1 associated signal in a particular pathway and in more than one gene. When SNPs were closely linked (R^2^>0.8), only one SNP was chosen for validation. Eight SNPs were genotyped in the *SLC23A1* (rs6596473, rs11950646) and *SLC23A2* (rs1715364), *MMP3* (rs679620), *MMP9* (rs3918241), *CYP19A1* (rs1870049, 2899472) and *ESR1* (3020314) genes (Table 4).

**Table 4:** Odds ratios (OR) and 95% confidence intervals (CI) for associated SNPs in the *SLC23A1*, *SLC23A2*, *MMP3*, *MMP9*, *CYP19A1 and ESR1* genes in the San Francisco Bay Area NHL and German studies.

	SNP and Genotype	All NHL	OR(95% CI)	DLBCL n(%)	OR(95% CI)	FL n(%)	OR(95% CI)	SLL n(%)	OR(95% CI)	Controls (US = 1049, GER = 494) %
	***SLC23A1*** ** (rs6596473)**									
US	GG	422 (44)	1.0	123 (46)	1.0	82 (41)	1.0	74 (49)	1.0	48
	CG	421 (44)	1.1 (.93–1.4)	122 (45)	1.1 (.85–1.5)	85 (43)	1.2 (.86–1.7)	58 (38)	.86 (.59–1.2)	42
	CC	114 (12)	1.2 (.91–1.6)	25 (9.0)	.91 (.57–1.5)	33 (17)	**1.8 (1.2–2.9)**	19 (13)	1.2 (.67–2.0)	11
	CG/CC	535 (56)	1.1 (.96–1.4)	147 (54)	1.1 (.83–1.4)	118 (59)	1.3 (.97–1.8)	77 (51)	0.92 (.65–1.3)	52
	*P for trend*		*0.11*		*0.90*		*0.01*		*1.0*	
Germany	GG	215 (46)	1.0	66 (46)	1.0	44 (56)	1.0	41 (43)	1.0	51
	CG	208 (45)	1.2 (.93–1.6)	71 (49)	1.0 (.60–1.7)	29 (37)	1.1 (.57–2.0)	41 (43)	1.0 (.58–1.8)	40
	CC	42 (9)	1.1 (.67–1.7)	8 (5)	.39 (.13–1.1)	6 (8)	1.2 (.37–4.0)	13 (14)	3.4 (.91–13)	9
	CG/CC	250 (54)	1.2 (.93–1.6)	79 (54)	.89 (.55–1.4)	35 (44)	1.1 (.61–2.0)	54 (57)	1.2 (.70–2.1)	49
	*P for trend*		*0.28*		*0.24*		*0.73*		*0.19*	
	***SLC23A1*** ** (rs11950646)**									
US	AA	380 (40)	1.0	112 (42)	1.0	75 (38)	1.0	65 (43)	1.0	43
	AG	436 (46)	1.1 (.92–1.3)	125 (47)	1.1 (.81–1.4)	84 (42)	1.1 (.79–1.6)	63 (42)	.91 (.62–1.3)	44
	GG	133 (14)	1.2 (.90–1.6)	31 (12)	.94 (.60–1.5)	39 (20)	**1.8 (1.2–2.8)**	22 (15)	1.2 (.68–1.9)	13
	AG/GG	569 (60)	1.1 (.94–1.3)	156 (58)	1.1 (.80–1.4)	123 (62)	1.3 (.92–1.7)	85 (57)	0.96 (.68–1.4)	57
	*P for trend*		*0.17*		*0.98*		*0.02*		*0.84*	
Germany	AA	198 (42)	1.0	63 (43)	1.0	37 (47)	1.0	36 (37)	1.0	46
	AG	225 (48)	1.2 (.91–1.6)	75 (51)	.90 (.54–1.5)	34 (44)	1.3 (.69–2.3)	46 (48)	1.3 (.74–2.4)	44
	GG	44 (10)	1.0 (.64–1.6)	9 (6)	.44 (.17–1.1)	7 (9)	1.9 (.55–6.7)	14 (15)	2.6 (.85–7.9)	10
	AG/GG	269 (58)	1.2 (.90–1.5)	84 (57)	.81 (.49–1.3)	41 (53)	1.4 (.76–2.4)	60 (63 )	1.5 (.82–2.6)	54
	*P for trend*		*0.47*		*0.15*		*0.24*		*0.09*	
	***SLC23A2*** ** (rs1715364)**									
US	TT	275 (29)	1.0	77 (28)	1.0	63 (32)	1.0	37 (25)	1.0	30
	CT	473 (49)	1.0 (.84–1.3)	145 (54)	1.1 (.83–1.5)	96 (48)	.91 (.64–1.3)	68 (45)	1.1 (.74–1.7)	51
	CC	210 (22)	1.2 (.95–1.6)	49 (18)	1.0 (.69–1.5)	41 (21)	1.1 (.70–1.7)	46 (30)	**1.9 (1.2–3.1)**	19
	CT/TT	680 (71)	1.1 (.89–1.3)	194 (72)	1.1 (.82–1.5)	137 (69)	0.95 (.69–1.3)	114 (76)	1.4 (.92–2.0)	70
	*P for trend*		*0.14*		.*78*		*0.86*		***6.4×10^−3^***	
										
Germany	TT	126 (28)	1.0	38 (27)	1.0	25 (32)	1.0	23 (24)	1.0	29
	CT	238 (52)	1.2 (.85–1.6)	72 (51)	.76 (.41–1.4)	41 (53)	1.2 (.59–2.4)	49 (52)	1.7 (.81–3.4)	46
	CC	92 (20)	.86 (.60–1.2)	30 (21)	.71 (.36–1.4)	11 (14)	.58 (.25–1.4)	23 (24)	1.8 (.77–4.1)	25
	TC/CC	330 (72)	1.0 (.78–1.4)	102 (73)	.74 (.42–1.3)	52 (68)	.95 (.50–1.8)	72 (76)	1.7 (.85–3.4)	71
	*P for trend*		*0.41*		*0.34*		*0.29*		*0.18*	
	***MMP3*** ** (rs679620)**									
US	AA	252 (26)	1.0	72 (27)	1.0	38 (19)	1.0	47 (32)	1.0	26
	AG	442 (46)	.93 (.75–1.2)	132 (49)	.97 (.70–1.3)	98 (49)	1.4 (.91–2.0)	64 (43)	.72 (.48–1.1)	49
	GG	260 (27)	1.0 (.82–1.3)	66 (24)	0.92 (.63–1.3)	64 (32)	**1.7 (1.1–2.6)**	38 (26)	.82 (.52–1.3)	26
	AG/GG	702 (74)	0.97 (.79–1.2)	198 (73)	0.95 (.70–1.3)	162 (81)	1.5 (1.0–2.2)	102 (68)	0.76 (.52–1.1)	74
	*P for trend*		*0.75*		*0.67*		***0.02***		*0.38*	
Germany	AA	121 (26)	1.0	42 (30)	1.0	24 (30)	1.0	22 (23)	1.0	28
	AG	235 (51)	1.1 (.85–1.6)	75 (53)	1.3 (.73–2.2)	38 (48)	.95 (.47–1.9)	50 (53)	1.2 (.60–2.4)	48
	GG	104 (23)	1.00 (.70–1.4)	25 (18)	.63 (.33–1.2)	17 (22)	.70 (.31–1.6)	23 (24)	1.3 (.60–2.9)	24
	AG/GG	339 (74)	1.1 (.83–1.5)	100 (70)	1.0 (.60–1.7)	55 (70)	.85 (.45–1.6)	73 (77)	1.3 (.65–2.4)	72
	*P for trend*		*0.93*		*0.20*		*0.42*		*0.48*	
	***MMP9*** ** (rs3918241)**									
US	TT	656 (69)	1.0	199 (74)	1.0	135 (68)	1.0	95 (63)	1.0	73
	AT	271 (28)	1.2 (.99–1.5)	64 (24)	0.94 (.69–1.3)	62 (31)	1.3 (.95–1.9)	50 (33)	1.6 (1.1–2.3)	25
	AA	30 (3.1)	1.7 (.95–3.0)	7 (2.6)	1.3 (.54–3.1)	4 (2.0)	1.1 (.36–3.1)	6 (4.0)	2.4 (.93–6.1)	2
	AT/AA	301 (31)	1.2 (1.0–1.5)	71 (26)	0.97 (.71–1.3)	66 (33)	1.3 (.95–1.8)	56 (37)	**1.6 (1.1–2.3)**	27
	*P for trend*		*0.01*		*0.99*		*0.15*		***4.8×10^−3^***	
Germany	TT	354 (76)	1.0	114 (78)	1.0	59 (75)	1.0	72 (76)	1.0	78
	AT	102 (22)	1.1 (.80–1.5)	28 (19)	.82 (.46–1.5)	19 (24)	1.2 (.54–2.5)	22 (23)	1.4 (.68–2.86)	21
	AA	10 (2)	2.5 (.80–8.1)	5 (3)	2.4 (.47–12.56)	1 (1)	-	1 (1)	1.2 (.07–19.49)	1
	AT/AA	112 (24)	1.1 (.84–1.5)	33 (22)	.92 (.54–1.6)	20 (25)	1.3 (.59–2.7)	23 (24)	1.4 (.68–2.8)	22
	*P for trend*		*0.24*		*0.91*		*0.47*		*0.40*	
	***CYP19A1*** ** (rs2899472)**									
US	CC	498 (52)	1.0	146 (54)	1.0	100 (50)	1.0	66 (44)	1.0	57
	AC	390 (41)	1.2 (1.0–1.5)	112 (41)	1.2 (.92–1.6)	80 (40)	1.3(.92–1.8)	67 (44)	**1.6 (1.1–2.3)**	36
	AA	71 (7.4)	1.1 (.81–1.6)	13 (4.8)	.72 (.39–1.3)	21 (10)	1.7 (1.0–2.9)	18 (12)	**2.2 (1.3–4.0)**	7
	AC/AA	461 (48)	1.2 (1.0–1.5)	125 (46)	1.1 (.87–1.5)	101 (50)	1.3 (.99–1.8)	85 (56)	**1.7 (1.2–2.4)**	43
	*P for trend*		*0.06*		*0.84*		***0.03***		***1.2×10^−3^***	
Germany	CC	276 (59)	1.0	88 (60)	1.0	49 (62)	1.0	55 (58)	1.0	53
	AC	157 (34)	.75 (.57–1.00)	49 (34)	.79 (.48–1.3)	23 (29)	.60 (.29–1.2)	36 (38)	.63 (.34–1.2)	41
	AA	32 (7)	1.0 (.60–1.8)	9 (6)	.78 (.32–1.9)	7 (9)	1.5 (.36–6.4)	4 (4)	1.1 (.24–5.1)	6
	AC/CC	189 (41)	.79 (.60–1.0)	58 (40)	.79 (.50–1.2)	30 (38)	.67 (.34–1.3)	40 (42)	.67 (.37–1.2)	47
	*P for trend*		*0.23*		*0.33*		*0.58*		*0.30*	
	***ESR1*** ** (rs3020314)**									
US	TT	435 (46)	1.0	122 (45)	1.0	112 (56)	1.0	62 (41)	1.0	43
	TC	423 (44)	0.93 (.78–1.1)	115 (43)	.90 (.68–1.2)	76 (38)	**.64 (.46–.88)**	68 (45)	1.1 (.74–1.5)	45
	CC	98 (10)	0.83 (.62–1.1)	33 (12)	.99 (.64–1.5)	13 (6)	**.42 (.23–.77)**	21 (14)	1.3 (.75–2.2)	12
	TC/CC	521 (55)	0.91 (.76–1.1)	148 (55)	.92 (.70–1.2)	89 (44)	**.59 (.44–.81)**	89 (59)	1.1 (.79–1.6)	57
	*P for trend*		*0.21*		*0.72*		***4.0×10^−4^***		*0.39*	
Germany	TT	221 (48)	1.0	67 (46)	1.0	43 (56)	1.0	49 (51)	1.0	48
	TC	205 (44)	1.0 (.78–1.4)	71 (48)	1.1 (.67–1.8)	30 (39)	**.47 (.22–1.0)**	37 (39)	.91 (.50–1.7)	44
	CC	38 (8)	1.0 (.62–1.7)	9 (6)	1.0 (.38–2.8)	4 (5)	**.24 (.06–.94)**	10 (10)	.95 (.36–2.5)	8
	TC/CC	243 (52)	1.0 (.79–1.3)	80 (54)	1.1 (.68–1.8)	34 (44)	**.42 (.20–.87)**	47 (49)	.92 (.51–1.6)	52
	P for trend		*0.87*		*0.77*		***0.01***		*0.82*	

1 ORs generated by conditional logistic regression.

The *SLC23A1* rs6596473 and rs11950646 SNPs associated with increased FL risk in the U.S. study (OR = 1.8, 95% CI = 1.2–2.9, *p* = 8.8×10^−3^ and OR = 1.8, 95% CI = 1.2–2.8, *p* = 8.3×10^−3^, respectively), did not influence risk of FL in the German study. However, in the German study, risk estimates were elevated for CLL (OR = 3.4, 95% CI = 0.91–13, *p* = 7.0×10^−2^; OR = 2.6, 95% CI = 0.85–7.9, *p* = 9.0×10^−2^, respectively) and ORs were reduced for DLBCL (OR = 0.39, 95% CI = 0.13–1.1, *p* = 8.0×10^−2^ and OR = 0.44, 95% CI = .17–1.1, *p* = 9.0×10^−2^, respectively). Consistent with the U.S. study, the *SLC23A2* rs1715364 CC genotype was associated with an elevated OR for SLL/CLL (U.S.: OR = 1.9, 95% CI = 1.2–3.1, *p* = 5.5×10^−3^; Germany: OR = 1.8, 95% CI = 0.77–4.1, *p* = 1.8×10^−1^), though the confidence interval included one.

For *MMP3* rs679620 where the US study found an increased risk for FL associated with the homozygous variant genotype (OR = 1.7, 95% CI = 1.1–2.6, *p* = 1.9×10^−2^), the German study found no associations with NHL. However, the homozygous variant genotype was associated with an increased risk for HL (OR = 2.3, 95% CI = 0.99–5.4, *p* = 5.4×10^−2^). Consistent with findings in the U.S. study, homozygosity for the *MMP9* rs3918241 variant resulted in an elevated OR for NHL approaching significance (US: OR = 1.7, 95% CI = 0.95–3.0, *p* = 7.4×10^−2^; Germany: OR = 2.5, 95% CI = 0.80–8.1; *p* = 1.0×10^−1^), however, there was no increased risk of CLL with the variant allele as found in the U.S. study.

In the German study, *CYP19A1* rs1870049 failed Pyrosequencing genotyping and no differences in rs2899472 allele frequencies were observed between cases and controls. The most robust findings were for *ESR1* rs3020314 where, as in the US study, heterozygous and homozygous variant genotypes were inversely related with FL risk in the German study (U.S.: heterozygous OR = 0.64, 95% CI = 0.46–0.88, *p* = 6.2×10^−3^; homozygous variant OR = 0.42, 95% CI = 0.23–0.77, *p* = 5.1×10^−3^; Germany: heterozygous OR = 0.47, 95% CI = 0.22–1.0, *p* = 5.7×10^−2^; homozygous variant OR = 0.24, 95% CI = 0.06–.094, *p* = 4.0×10^−2^). The *ESR1* rs3020314 variant allele also was associated with HL in the German study (OR = 0.61, 95% CI = 0.35–1.1, *p* = 7.7×10^−2^).

## Discussion

Here, we report that SNPs in the vitamin C receptor and matrix metalloproteinase gene families and in genes involved in estrogen responsiveness may be risk alleles for NHL. Our main strategy was to use a hypothesis-driven approach to further examine genetic findings based on our earlier studies by genotyping SNPs in related pathways or in genes that share common biological functions to those previously associated with NHL [2], [4]–[12]. We selected potential functionally relevant SNPs including non-synonymous coding SNPs, and 1–15 haplotype tagging SNPs were genotyped based on known patterns of LD using data available from the HapMap CEU population. These tag SNPs captured an average 39% of all common SNPs (R^2^>0.8) in the studied genes and their flanking regions. We note that the low coverage in some genes may have precluded us from detecting additional NHL risk alleles. Analyses were restricted to white non-Hispanics with ancestry data to reduce population stratification. In 959 cases and 1,049 controls, we identified 14 unique SNPs in eight pathways and seven haplotypes associated with NHL after correction for multiple testing at the adjusted *q*<0.10 (estimated FDR) level, and seven SNPs and eight haplotypes remained significantly associated with lymphoma risk at the *q*<0.05 level. We utilized *in silico* algorithms to predict likely functional effects for associated SNPs. Next, in an independent case-control study of lymphoma, we performed a small replication analysis of associated SNPs that were chosen based on the criteria that multiple SNP-NHL associations were observed in the same gene or gene family. In doing so, we found a strong inverse association between FL and an intronic SNP in *ESR1* in both study populations. Other associations were observed between risk of lymphoma subtypes and *SLC23A1*, *SLC23A2*, and *MMP9* SNPs. Although several of these associations occurred within different NHL subtypes across the two study populations, the overall consistency of associations with specific genes provides some support that these genes might be associated with NHL risk. Replication in additional populations and investigation of molecular similarities and differences of NHL subtypes such as through tumor microarray studies will be needed to clarify these findings.

### Vitamin C and risk of NHL

The associations we identified between NHL and SNPs in the vitamin C receptor genes may provide indirect evidence to support epidemiological data showing inverse associations between NHL risk and intakes of fruits and vegetables, which are primary sources of dietary vitamin C [14], [15]. Here, we observed that homozygous variant genotypes for two closely linked intronic *SLC23A1* SNPs, rs6596473 and rs11950646, were positively associated with FL in the U.S. study. However, in the German study, these genotypes were inversely and positively associated with DLBCL and SLL/CLL risk, respectively, suggesting that further study will be needed to determine the relevance, if any, of these findings. In the U.S. analysis of seven *SLC23A2* SNPs, we found that variant alleles for rs6133175 and rs1715364 increased SLL/CLL risk and rs1776948 for FL and SLL/CLL. In genotyping *SLC23A2* rs1715364 in the German study, the variant CC genotype was associated with an elevated OR for CLL, consistent with findings from the U.S. study. The haplotype analyses supported the genotype results but added nothing further to the individual SNP associations.

*SLC23A1* and *2* encode two vitamin C transport proteins, SVCT1 and 2, respectively[16] that exhibit non-redundant functions[17]. The lethality of *slc23a1* and *slc23a2* mouse knockouts highlights the relevance of these proteins in maintaining vitamin C homeostasis [17], [18]. In humans, SVCT1 is expressed in kidney, intestinal, hepatic and placental tissues and is critical for vitamin C absorption and reabsorption, whereas SVCT2 is expressed in most tissues and is essential for vitamin C bioaccumulation[19]. Thus, the influence of *SLC23A1* and *SLC23A2* genetic variation on NHL risk suggests that both vitamin C uptake and storage may be involved in the pathogenesis of lymphoma. *In silico* models suggest that the *SLC23A2* rs1715364 T allele may augment *SLC23A2* expression and increase vitamin C bioavailability, which will need to be tested in functional studies. By extension, if the variant C allele results in lower *SLC23A2* expression, then the increased NHL risk associated with the variant C allele might suggest that reduced vitamin C bioavailability increases lymphoma risk.

As with the majority of SNPs investigated in this study, *SLC23A1* and *SLC23A2* SNPs were chosen as tag SNPs to gain gene coverage to capture potential causal variants. Though *SLC23A1* and *SLC23A2* have extensive sequence homology, *SLC23A2* is a 10-fold larger gene with a much more complex locus architecture (Figure S1). Whereas, two *SLC23A1* tag SNPs captured 75% of its haplotype structure, seven *SLC23A2* tag SNPs captured only 35% of its haplotype structure at an R^2^>0.94, warranting the need for additional fine mapping of the *SLC23A2* gene.

In pooled analyses of two NHL case-control studies, we previously reported that SNPs that impair anti-oxidant capacity may influence lymphomagenesis[5]. Vitamin C is an essential enzyme cofactor in reactions catalyzed by several metal-dependent oxygenases[20]. It also functions as a scavenger of reactive oxygen species[21] and, thus, may play an important role in preventing oxidative stress induced DNA damage by quenching free radical formation. Other possible modes of action may involve vitamin C's anti-tumorigenic role in supporting proper collagen formation and matrix stabilization[22]. Further, in an *in vitro* study of T-cell leukemia, vitamin C abrogated cell proliferation and induced apoptosis through p53, p21, Bcl-2 and Bax modulation[23]. Additional studies will be needed to clarify the role of vitamin C in the pathogenesis of NHL.

### MMP SNPs influence NHL, FL and SLL/CLL risk

Extracellular matrix degradation is involved in many normal physiological processes including development, growth and tissue repair. However, high MMP-3 and -9 proteolytic enzyme levels also may support tumor cell proliferation, angiogenesis, invasion and metastasis[24], [25] by degrading type IV collagen, penetrating the basement membrane and invading surrounding tissues[26]. High MMP expression is found in lymphoid cells and malignantly transformed cells[27]. Recent studies highlight the relevance of MMPs and angiogenesis in lympho-hematological malignancies such as B-cell lymphoma, multiple myeloma, CLL and acute lymphocytic and myeloid leukemias[28]. While the MMP family has been related to cancer invasion and metastases, our study suggests that variation in *MMP* genes also plays a role in lymphomagenesis. Here we found that homozygosity for a non-synonymous *MMP3* 45K>E SNP, rs679620, was associated with increased risk of FL in the U.S. study, and for HL in the German study. It is biologically feasible that the *MMP3* 45EE allelotype may influence lymphomagenesis since it is predicted by RESCUE ESE to enhance exon 2 splicing, which may increase *MMP3* expression. In the U.S. study, increased FL and SLL/CLL risk also was found in those possessing variant alleles for an *MMP9* promoter SNP, rs3918241, and two non-synonymous *MMP9* SNPs, rs17576 (279Q>R) and rs2274756 (668R>Q). According to ESEfinder, the rs3918241 variant A allele may enhance *MMP9* promoter activity and the rs2274756 variant 668Q allelotype creates an extra binding motif that may enhance exon 2 splicing. Thus, the rs3918241 and rs2274756 risk alleles for SLL/CLL are predicted to increase *MMP9* expression. We tested the association of rs3918241 in the German study and found an elevated though non-significant increase in NHL risk, consistent with the U.S. study, though there was no evidence of an association with FL and CLL as in the U.S. study. The 4% frequency of the homozygous variant genotype may have precluded us from observing these associations, which will require the study of this SNP in larger populations. Since rs3918241 and rs2274756 are in complete LD, additional studies also will be needed to discern which, if any, of these functionally relevant SNPs may be causally related to NHL.

In earlier studies, we found associations with NHL and SNPs in energy regulation genes such as leptin (*LEP*), leptin receptor (*LEPR*) and ghrelin (*GHRL*)[9], [11], [12]. We hypothesized that leptin may promote lymphomagenesis through mitogenic and anti-apoptotic effects on B-cell populations through *LEPR*-mediated upregulation of BCL-2, MMPs, and tissue inhibitors of metalloproteinases. The present study lends further credence to the role of MMPs in lymphomagenesis. Interestingly, our study found multiple genetic associations with *LEPR*, *GHRL* and SNPs in other energy regulatory genes, though the adjusted *q*-values were >0.10. In light of these findings, we plan to further study the relationship between energy regulation and *MMP* SNPs in lymphomagenesis.

### ESR1 and CYP19A1 gene variants in association with lymphoma risk

Previously, we reported a *CYP17A1*−34T>C high-risk genotype for DLBCL in two independent case-control studies of NHL[7], [10]. *CYP17A1* produces key enzymes that catalyze the synthesis of estrone and dehydroepiandrosterone, the precursors for estrogen and testosterone. These findings prompted us to explore SNPs in additional genes involved in steroidogenesis and in estrogen and testosterone uptake and metabolism. Unfortunately, in the present study, we were unable to obtain genotype calls for *CYP17A1*−34T>C due to poor genotype clustering. Additionally, we had insufficient SNP coverage across *ESR1* since some tags were located in the lower LD regions (Figure S4). Nonetheless, in the U.S. study, our results revealed associations with NHL and multiple SNPs in the *CYP19A1* and *ESR1* genes. *CYP19A1* encodes for aromatase, a key enzyme in estrogen production[29] that converts androstenedione and testosterone to estradiol and estrone, respectively. *ESR1* encodes estrogen receptor 1 (ERα) that plays an important role in mediating estrogen action in target tissues. Moreover, ERα signaling regulates estrogen production by direct modulation of the *CYP19A1* promoter[30]. Here we found seven *CYP19A1* SNPs associated with forms of NHL and one, rs1870046, remained associated with FL (*q*<0.10). In the replication study, rs1870046 was not successfully genotyped and no associations were found between SLL/CLL and rs2899472. Given the extent of associated alleles in the U.S. study and that only one SNP was investigated in the German study, further investigation of associated alleles in the *CYP19A1* gene is warranted.

The most significant finding in this study was the inverse association between FL and the C allele in *ESR1* rs3020314 that was corroborated with FL and HL in the German study. Since estrogen exerts a biphasic dose-dependent effect on the immune system, this association may be biologically plausible. Notable but somewhat complex hormonal-driven associations have been reported between other *ESR1* gene variants and breast cancer, spontaneous abortion, osteoporosis[31], age of menarche[32], genital abnormalities in men[33] and fertility in men and women. *ESR1* is also expressed by thymocytes and B- and T-cells[34] and *Esr1* knockout mice exhibit compromised thymic[35] and B-cell development[36], insulin resistance, impaired glucose tolerance and obesity[37]. Several epidemiological studies have reported that oral contraceptive use and hormone replacement therapy are associated with lower lymphoma risk [7], [38], [39]. Thus, large pooled gene-environment studies of *ESR1* gene variants and measures of hormone levels or history of hormone use may be useful to further explore the role of hormones in the pathogenesis of lymphoma.

### Lack of additional genetic associations

Few, if any, additional SNP and haplotype associations were observed in other pathways investigated in this study. For instance, *BIK* and *EPHX2* were the only genes associated with NHL in the immunity and xenobiotic metabolism/oxidative stress pathways, respectively, and no NHL-genetic associations were observed in the sunlight/vitamin D or folate metabolism pathways. Previous studies suggest that SNPs in these pathways may influence lymphoma risk [2]. Our inability to validate these associations here may be that our study was underpowered to detect significant associations after adjusting for multiple comparisons; that our study had incomplete gene coverage; or that other genetic mechanisms such as copy number variation influenced the results. Thus, negative results reported here do not dismiss the potential relevance of these SNPs/pathways in lymphomagenesis, which will require further study in larger populations.

### Perspectives and Future Directions

This study reports novel associations with common variants in *ESR1* and in the vitamin C receptor and matrix metalloproteinase gene families. Of particular importance is the replication of a significant association with FL and the *ESR1* rs3020314 SNP in a second independent study of lymphoma, which suggests that estrogen bioavailability may be relevant in the pathogenesis of lymphoma. We also believe that the genetic associations that did not reach statistical significance in the replication study are noteworthy, but due to the sample size, may have been underpowered to detect subtle allelic associations. Given the fact that we adjusted for multiple comparisons for pathways and did not adjust for all comparisons with all NHL subtypes, there still is an increased chance for type 1 errors and it is possible that some of our results are false positive associations. These findings will need further validation in larger studies. Owing to the small number of SNPs analyzed in the replication study, investigation of associated SNPs identified in the U.S. population will be ongoing. Further replication, fine mapping and functional studies will also be needed to determine the causal variants, and pooled data from consortium studies will be necessary to discern more modest effects of low penetrance SNPs or for rare alleles. The verification of these findings could have important implications by providing mechanistic clues in the etiology of lymphoma and in identifying useful therapeutic targets. Interestingly, most associations were found for FL and SLL/CLL and few were identified for DLBCL. This may be due to the heterogeneity of the disease since GC- and post-GC types of DLBCL exhibit significant molecular diversity at the gene expression and phenotype levels [45]–[47] that may require further stratification to detect underlying DLBCL risk alleles. It may also be that our hypothesis-driven approach failed to interrogate the appropriate pathways. To overcome this, we are currently performing a whole genome scan that will potentially lead to the identification of novel DLBCL susceptibility alleles.

## Materials and Methods

### U.S. Study Population

Cases were identified within ∼1 month of diagnosis by the Northern California Cancer Center's rapid case ascertainment. For this interim analysis which includes only a subset of the study participants, eligible patients were diagnosed with incident NHL from 10/2001–10/2005. All eligible patients were 20–84 years old and residents of one of six Bay Area counties at the time of diagnosis. There were 1704 eligible cases interviewed between 10-2001 and 10-2005. An additional 452 cases died prior to initial contact, 272 could not complete an interview in English, 70 had physician indicated contraindications to contact, 161 could not be located/moved, 155 were too ill and 360 refused to participate. Pathology reports and diagnostic materials were obtained from the diagnosing pathology department and re-reviewed by an expert pathologist to confirm the diagnosis and to provide a consistent classification of NHL subtypes using the REAL/WHO[40] lymphoma classification. Control participants were identified by random digit dial supplemented by random sampling of Centers for Medicare & Medicaid Services lists for those ≥65 years old. Eligibility criteria for controls were identical to cases with the exception of NHL diagnosis. Control participants were frequency matched to cases by age within five years, sex and county of residence. Among 1939 eligible random-digit-dial controls, 1313 completed in-person interviews, 85 were too ill and 541 refused to participate. Among 1475 eligible Medicare controls, 768 completed in-person interviews, 97 were too ill and 610 refused to participate. Demographic and risk factor data including HIV status, self-reported race and ethnicity, and paternal and maternal ancestry, were collected from all study participants during in-person interviews. No proxy interviews were conducted. The median time from diagnosis to venipuncture was 6.5 months. Blood and/or buccal cell specimens were collected for eligible participants who agreed to biospecimen collection (87% of cases, 88% of controls).

### German Study Population

Detailed information regarding the design of this population-based case-control study has been published elsewhere[41]. Briefly, the study was carried out from 1999–2002 in six regions of Germany among 18–80 year-old adults. The study included 710 cases of DLBCL, FL, CLL, multiple myeloma (MM), MALT lymphoma, T-cell lymphoma and Hodgkin lymphoma (HL) and 710 controls individually matched by sex, age (+/−1 year of birth) and study region. Of these, there were 494 NHL cases and 111 HL cases and matched controls. The median time from diagnosis to blood collection was 27 days and the response rate for cases and controls was 87% and 44%, respectively. Analyses for all NHL did not include MM or HL. For subtype-specific analyses, only FL, DLBCL, CLL and HL were considered. Incident lymphoma cases were recruited from hospitals and office-based physicians involved in diagnosis and treatment of lymphoma in the study regions, interviewed by trained interviewers and provided blood samples. Cases were classified according to the WHO system based on pathology reports and 20% of cases were re-reviewed by a team of lymphoma pathologists.

The U.C. San Francisco Committee on Human Research, the U.C. Berkeley Committee for Protection of Human Subjects, and the Ethics Committee, European Union, approved study protocols. All study participants provided written informed consent prior to interview and biospecimen collection.

### Pathway, Gene and SNP Selection

Eight pathways or groups of genes with common biological function in a total of 146 genes (Table 1) were chosen to investigate *a priori* hypotheses (Table S5) in steroidogenesis, immune function, cell signaling, sunlight exposure/vitamin D, xenobiotic metabolism/oxidative stress, energy balance, cholesterol synthesis/metabolism and diet (folate and vitamin C uptake and/or metabolism) pathways. 1–15 SNPs in each gene and a total of 768 SNPs were selected using a minimum minor allele frequency criterion of ≥10%, based upon HapMap data release 12 (∼October 2004) for CEU Caucasian samples. Additional coding and regulatory SNPs, especially those with non-synonymous amino acid changes, were identified using Ensembl, NCBI, and SNPper databases [42]–[47]. For all genes, haplotype tagging SNPs were selected using Haploview[48] after downloading SNP genotyping information available for the CEPH population (Utah residents with ancestry from northern and western Europe) from the HapMap database (Build 34)[43], [49]. SNP validation scores between 0 and 1 were generated using the Illumina Assay Design Tool to estimate the likelihood that SNPs would be successfully genotyped. Through an iterative process, we dropped SNPs with validation scores <0.6 and replacement haplotype tagging SNPs were identified using Haploview until a complete panel of 768 SNPs were chosen. This SNP panel (GS0006402-OPA) was custom designed by Illumina.

### Genotyping

For the U.S. study, genotyping was performed using the Illumina GoldenGate assay on a BeadStation 500G Genotyping System. Genotype calls were made using the GenCall version 6.2.0.4 software package (Illumina, Inc., San Diego, CA). Summary files were generated using the GTS Reports version 5.1.2.0. All SNPs were examined for cluster separation using Illumina quality scores that were automatically generated by the software. Poorly performing loci were excluded, designated with a GenTrain score <0.4 or a cluster separation score <0.6. SNPs were further excluded for scattered or overlapping clusters, for controls not in Hardy-Weinberg equilibrium, for low frequency (<0.05), or for DNA samples with low intensity. Individual DNA samples were excluded from the study if there was no signal across SNPs. Genotyping of the German study was performed by Pyrosequencing™ (Biotage, Uppsala, Sweden)[50].

#### Quality assurance

To validate genotyping results, each 96-well plate contained 90 randomized study samples, two randomized replicate study samples placed side-by-side and three laboratory control DNA samples. DNA also was genotyped from six CEPH families (Coriell Institute, Camden, NJ). Genotyping of 10 SNPs was performed for validation purposes using TaqMan® genotyping assays and the ABI Prism 7700 Sequence Detection System (Applied Biosystems, Foster City, CA).

#### Coverage estimation

Coverage which indicates the fraction of common HapMap markers successfully tagged by the set of genotyped SNPs was estimated using the pairwise tagging method in Haploview[48], which is based on the Tagger algorithm (www.broad.mit.edu/mpg/tagger/). SNPs genotyped were forced into a tag list and neighboring HapMap SNPs were explicitly prohibited from being chosen as tags. We specified that all HapMap markers being captured by the set of tags should be correlated at R^2^≥0.8 with at least one marker in the set. HapMap markers with MAF<0.05 were excluded.

### Statistical Analyses

All statistical analyses were restricted to HIV-negative participants of white non-Hispanic origin (959 cases, 1,049 controls). Because self identified ethnicity and ancestry correlates very well with genetic structure [51], using this information greatly minimizes the potential effect of underlying population stratification on the risk estimates. Hardy-Weinberg equilibria for genotype frequencies among controls were determined by exact tests using a Markov-chain method (Genepop v3.4).

#### SNP analysis

Exact tests were used to generate *p*-values to test associations of case-control status and SNP associations. For each pathway, *P*-values were corrected for multiple comparisons (*q*-values) using the false discovery rate[52]. For all NHL, and for each subtype tested, the following total number of SNP tests were performed in each pathway: steroidogenesis, 88; immunity, 75; cell signaling, 88; sunlight/vitamin D, 53; xenobiotic/metabolism, 40; energy balance, 155; cholesterol, 62; and diet, 26. Fifty intergenic SNPs were excluded from the pathway analyses. A threshold of *q*<0.10 was used in reporting the most relevant findings in Tables 2 and 3. ORs and 95% CIs as estimates of the relative risk were computed adjusted by age and sex from unconditional logistic regression [53] without taking into account multiple testing. In genotype analyses, the wild-type category (chosen either as the most common homozygous genotype or arbitrarily if the same) was the reference group. The odds ratios from the unconditional logistic models were adjusted for the frequency-matched variables age in 5-year groups and sex. Analyses were performed on all NHL cases and then stratified by DLBCL, FL, SLL/CLL and “others.” We did not adjust for all multiple comparisons for all SNPs in all types of lymphoma.

#### Gene analysis

We tested for multi-way interactions within sets of SNPs spanning a gene by fitting the genotypes to a linear regression model and applying a t-test to the case-control distributions. *P*-values were generated by permuting cases and controls, repeating the experiment 10,000 times per gene. Results were adjusted for multiple hypotheses testing using the FDR for each functional group/pathway. For “all NHL” and for each subtype tested, the following total number of gene tests were performed: steroidogenesis, 22; immunity, 22; cell signaling, 19; sunlight/vitamin D, 12; xenobiotic/metabolism, 14; energy balance, 38; cholesterol, 19; and diet, 9.

#### Haplotype analysis

SNPs were partitioned into blocks of limited diversity using HAP[54], which uses criteria similar to previous methods[55]. For each block of limited diversity, genotypes were phased using PHASE[56]. For each common haplotype (MAF>5%), a χ^2^-test with one degree of freedom was performed with counts of haplotype occurrences in cases and controls. Results were adjusted for multiple hypotheses using the FDR for each functional group/pathway. For “all NHL” and for each subtype tested, the following total number of haplotype tests were performed: steroidogenesis, 44; immunity, 38; cell signaling, 45; sunlight/vitamin D, 31; xenobiotic/metabolism, 19; energy balance, 91; cholesterol, 36; and diet, 12.

#### Replication study

Conditional logistic regression analysis was used to estimate ORs and 95% CIs comparing cases (all NHL cases combined –which excluded MM and HL- and individual FL, DLBCL and SLL/CLL subtypes and separate analyses for HL) to respective controls in association with genotypes, adjusting for gender, age and study region.

#### In silico algorithms

The FASTSNP web server[57] was used to interrogate 11 external web servers including RESCUE-ESE[13], ESEfinder[58] and PolyPhen[59] to predict the functionality of SNPs.

## Supporting Information

Figure S1 LD plot characterizing haplotype blocks in SCL23A2. The 7 SLC23A2 SNPs genotyped (rs2298174, rs1629176, rs1715385, rs6133175, rs1715364, rs1776948 and rs6139587 highlighted in green) captured 35% of genetic variation for the SLC23A2 locus. Detailed description of LD plots shown in Figures S1, S2, S3 and S4. R2 values are indicated in percentages within squares in the LD plot, where red indicates regions of high LD and white represents regions of low LD. Blocks without numbers indicate r2 = 1 (100%). At the top, figures display a track with HapMap genotyped SNPs and genes within the genomic region. SNPs that have been genotyped in this study are highlighted in green and marked with lines from the SNP position to the LD chart. LD blocks were generated using the Confidence Intervals method included in Haploview4.0 (http://www.broad.mit.edu/mpg/haploview/index.php), that creates 95% confidence bounds on D' considered to be in strong LD where 95% of the comparisons made are informative. Genotype data was retrieved from HapMap release 22/phaseII (NCBI B36 assembly), for the CEPH (Centre d'Etude du Polymorphisme Humain) from Utah (CEU). Coverages were obtained using the tagging method included in Haploview as described in the manuscript.
(59.29 MB DOC)

Figure S2 LD plot characterizing haplotype blocks in MMP9. The 5 MMP9 SNPs genotyped (rs6094237, rs4810482, rs3918241, rs17576 and rs2274756), highlighted in green, captured 15 out of 19 variations (78%) with r2>0.8. There is, therefore, good coverage of the MMP9 gene.
(2.58 MB DOC)

Figure S3 LD plot characterizing haplotype blocks in CYP19A1. The 14 CYP19A1 SNPs genotyped (rs10046, rs2289105, rs2899472, rs9944225, rs4775936, rs2008691, rs4774584, rs7181886, rs936306, rs1004982, rs2470146, rs2470144, rs1870049 and rs752760 highlighted in green) captured 66% of genetic variation for CYP19A1.
(72.13 MB DOC)

Figure S4 LD plot characterizing haplotype blocks in ESR1. The 11 ESR1 SNPs genotyped (rs2881766, rs488133, rs532010, rs827423, rs1709182, rs1913474, rs3020314, rs722208, rs3020411, rs2813545 and rs910416 highlighted in green) captured 66 out of 262 SNPs at r2>0.8, providing 25% gene coverage.
(70.04 MB DOC)

Table S1 Odds ratios (OR) and 95% confidence intervals (CI) for associated SLC23A1 and SLC23A SNPs in the San Francisco Bay Area NHL study.
(0.11 MB DOC)

Table S2 Odds ratios (OR) and 95% confidence intervals (CI) for associated MMP1, 3 and 9 SNPs in the San Francisco Bay Area NHL study.
(0.14 MB DOC)

Table S3 Odds ratios (OR) and 95% confidence intervals (CI) for associated CYP19A1 SNPs in the San Francisco Bay Area NHL study.
(0.16 MB DOC)

Table S4 Odds ratios (OR) and 95% confidence intervals (CI) for associated ESR1 SNPs in the San Francisco Bay Area NHL study.
(0.12 MB DOC)

Table S5 A priori hypotheses tested.
(0.06 MB DOC)
