# Supplementary figures and images for: Polymorphisms in the Estrogen Receptor 1 and Vitamin C and Matrix Metalloproteinase Gene Families Are Associated with Susceptibility to Lymphoma
Source: PLoS One. 2008 Jul 30;3(7):e2816. doi: 10.1371/journal.pone.0002816 (PMC2474696; doi:10.1371/journal.pone.0002816)

**
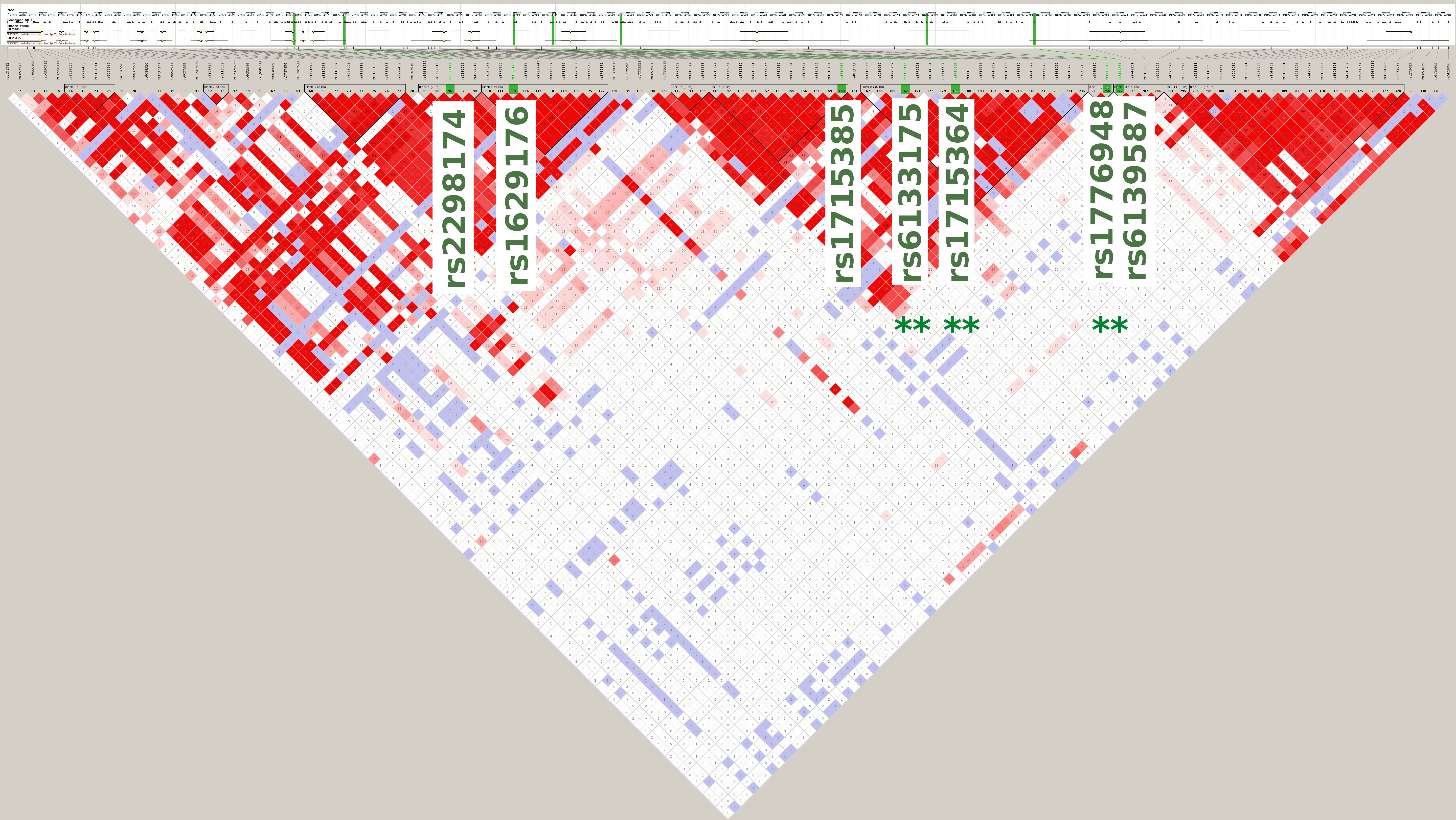
**

Supplement: Figure S1 — LD plot characterizing haplotype blocks in SCL23A2. The 7 SLC23A2 SNPs genotyped (rs2298174, rs1629176, rs1715385, rs6133175, rs1715364, rs1776948 and rs6139587 highlighted in green) captured 35% of genetic variation for the SLC23A2 locus. Detailed description of LD plots shown in Figures S1, S2, S3 and S4. R2 values are indicated in percentages within squares in the LD plot, where red indicates regions of high LD and white represents regions of low LD. Blocks without numbers indicate r2 = 1 (100%). At the top, figures display a track with HapMap genotyped SNPs and genes within the genomic region. SNPs that have been genotyped in this study are highlighted in green and marked with lines from the SNP position to the LD chart. LD blocks were generated using the Confidence Intervals method included in Haploview4.0 (http://www.broad.mit.edu/mpg/haploview/index.php), that creates 95% confidence bounds on D' considered to be in strong LD where 95% of the comparisons made are informative. Genotype data was retrieved from HapMap release 22/phaseII (NCBI B36 assembly), for the CEPH (Centre d'Etude du Polymorphisme Humain) from Utah (CEU). Coverages were obtained using the tagging method included in Haploview as described in the manuscript. (59.29 MB DOC) [file pone.0002816.s001.doc]

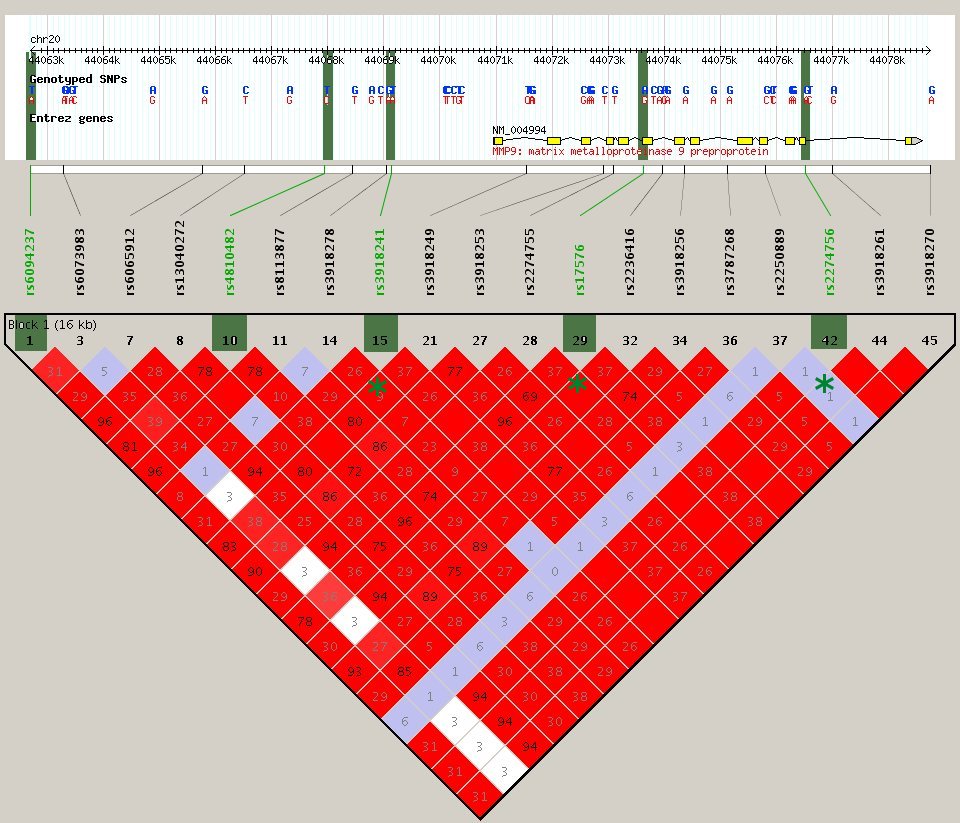

Supplement: Figure S2 — LD plot characterizing haplotype blocks in MMP9. The 5 MMP9 SNPs genotyped (rs6094237, rs4810482, rs3918241, rs17576 and rs2274756), highlighted in green, captured 15 out of 19 variations (78%) with r2>0.8. There is, therefore, good coverage of the MMP9 gene. (2.58 MB DOC) [file pone.0002816.s002.doc]

**
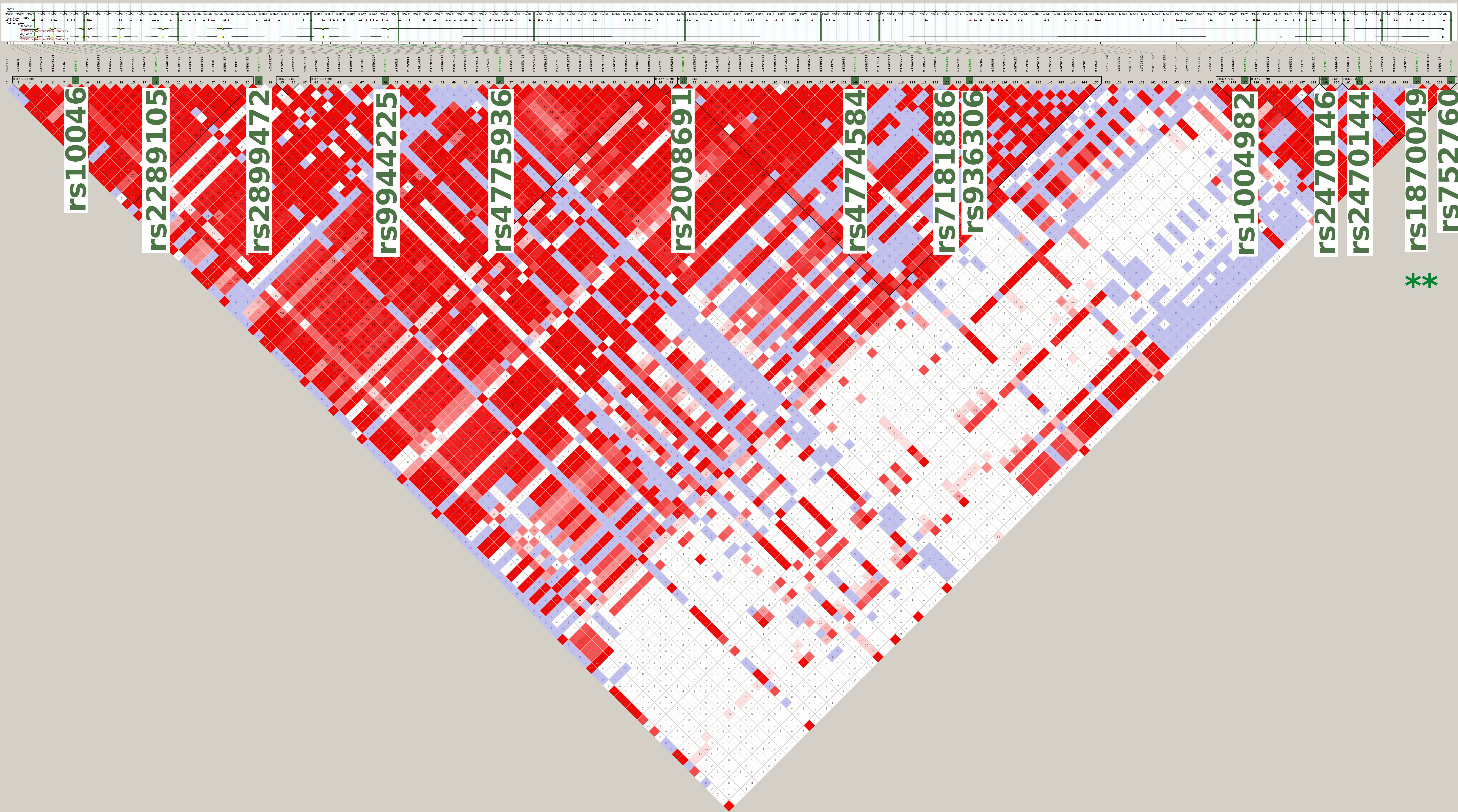
**

Supplement: Figure S3 — LD plot characterizing haplotype blocks in CYP19A1. The 14 CYP19A1 SNPs genotyped (rs10046, rs2289105, rs2899472, rs9944225, rs4775936, rs2008691, rs4774584, rs7181886, rs936306, rs1004982, rs2470146, rs2470144, rs1870049 and rs752760 highlighted in green) captured 66% of genetic variation for CYP19A1. (72.13 MB DOC) [file pone.0002816.s003.doc]

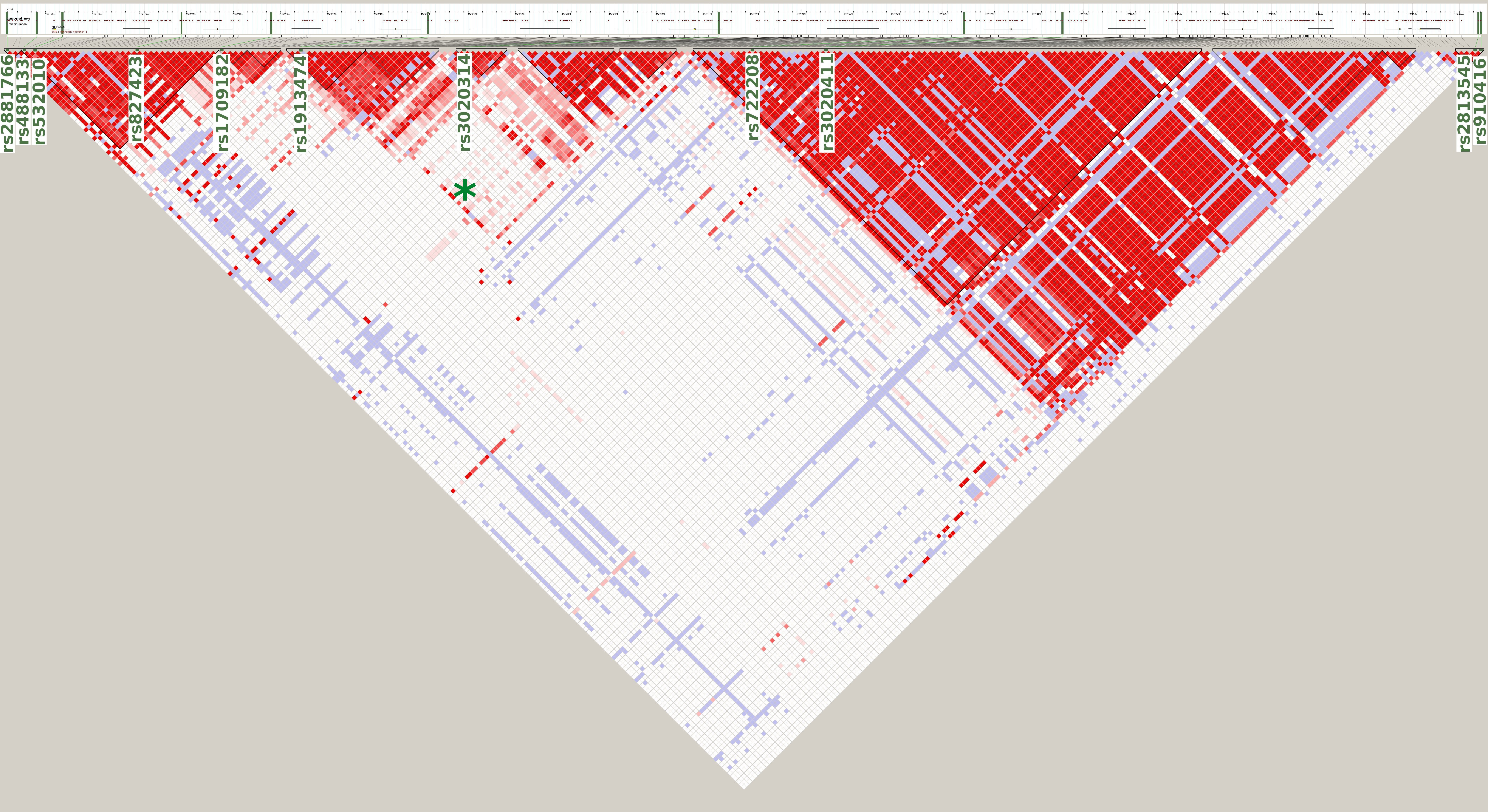

Supplement: Figure S4 — LD plot characterizing haplotype blocks in ESR1. The 11 ESR1 SNPs genotyped (rs2881766, rs488133, rs532010, rs827423, rs1709182, rs1913474, rs3020314, rs722208, rs3020411, rs2813545 and rs910416 highlighted in green) captured 66 out of 262 SNPs at r2>0.8, providing 25% gene coverage. (70.04 MB DOC) [file pone.0002816.s004.doc]
