# Supplementary material for: Polymorphisms in the Estrogen Receptor 1 and Vitamin C and Matrix Metalloproteinase Gene Families Are Associated with Susceptibility to Lymphoma
Source: PLoS One. 2008 Jul 30;3(7):e2816. doi: 10.1371/journal.pone.0002816 (PMC2474696; doi:10.1371/journal.pone.0002816)
Supplement: Table S1 — Odds ratios (OR) and 95% confidence intervals (CI) for associated SLC23A1 and SLC23A SNPs in the San Francisco Bay Area NHL study. (0.11 MB DOC) [file pone.0002816.s005.doc]

**Table S1. Odds ratios (OR) and 95% confidence intervals (CI) for associated *SLC23A1 and SLC23A*** SNPs in the San Francisco Bay Area NHL study.

| SNP & Genotype | All NHL (N=959) n(%) | OR(95% CI) | DL (N=271) n(%) | OR(95% CI) | FL (N=201) n(%) | OR(95% CI) | SLL (N=151) n(%) | OR(95% CI) | Other (N=334) n(%) | OR(95% CI) | Controls (N=1049) n(%) |
| --- | --- | --- | --- | --- | --- | --- | --- | --- | --- | --- | --- |
| SLC23A1 |  |  |  |  |  |  |  |  |  |  |  |
| **rs6596473** |  |  |  |  |  |  |  |  |  |  |  |
| GG | 422 (44) | 1.0 | 123 (46) | 1.0 | 82 (41) | 1.0 | 74 (49) | 1.0 | 143 (43) | 1.0 | 496 (48) |
| CG | 421 (44) | 1.1 (.93-1.4) | 122 (45) | 1.1 (.85-1.5) | 85 (43) | 1.2 (.86-1.7) | 58 (38) | 0.86 (.59-1.2) | 156 (47) | 1.2 (.94-1.6) | 438 (42) |
| CC | 114 (12) | 1.2 (.91-1.6) | 25 (9.0) | 0.91 (.57-1.5) | 33 (17) | **1.8 (1.2-2.9)** | 19 (13) | 1.2 (.67-2.0) | 35 (10) | 1.1 (.72-1.7) | 110 (11) |
| CG/CC | 535 (56) | 1.1 (.96-1.4) | 147 (54) | 1.1 (.83-1.4) | 118 (59) | 1.3 (.97-1.8) | 77 (51) | 0.92 (.65-1.3) | 191 (57) | 1.2 (.93-1.5) | 548 (52) |
| *p for trend* |  | *0.11* |  | *0.90* |  | ***0.01*** |  | *1.0* |  | *0.30* |  |
| **rs11950646** |  |  |  |  |  |  |  |  |  |  |  |
| AA | 380 (40) | 1.0 | 112 (42) | 1.0 | 75 (38) | 1.0 | 65 (43) | 1.0 | 128 (39) | 1.0 | 447 (43) |
| AG | 436 (46) | 1.1 (.92-1.3) | 125 (47) | 1.1 (.81-1.4) | 84 (42) | 1.1 (.79-1.6) | 63 (42) | 0.91 (.62-1.3) | 164 (50) | 1.2 (.94-1.6) | 460 (44) |
| GG | 133 (14) | 1.2 (.90-1.6) | 31 (12) | 0.94 (.60-1.5) | 39 (20) | **1.8 (1.2-2.8)** | 22 (15) | 1.2 (.68-1.9) | 39 (12) | 1.0 (.68-1.5) | 132 (13) |
| AG/GG | 569 (60) | 1.1 (.94-1.3) | 156 (58) | 1.1 (.80-1.4) | 123 (62) | 1.3 (.92-1.7) | 85 57) | 0.96 (.68-1.4) | 203 (61) | 1.2 (.92-1.5) | 592 (57) |
| *p for trend* |  | *0.17* |  | *0.98* |  | ***0.02*** |  | *0.84* |  | *0.46* |  |
| SLC23A2 |  |  |  |  |  |  |  |  |  |  |  |
| **rs2298174** |  |  |  |  |  |  |  |  |  |  |  |
| GG | 457 (48) | 1.0 | 115 (43) | 1.0 | 107 (54) | 1.0 | 80 (53) | 1.0 | 153 (46) | 1.0 | 517 (50) |
| CG | 415 (43) | 1.1 (.93-1.3) | 134 (50) | 1.4 (1.1-1.9) | 73 (37) | 0.85 (.62-1.2) | 59 (39) | 0.88 (.61-1.3) | 149 (45) | 1.2 (.92-1.5) | 419 (40) |
| CC | 83 (8.7) | 0.85 (.62-1.2) | 21 (7.8) | 0.85 (.51-1.4) | 20 (10) | 0.90 (.53-1.5) | 11 (7.3) | 0.61 (.31-1.2) | 31 (9.3) | 0.93 (.60-1.4) | 109 (10) |
| CG/CC | 498 (52) | 1.1 (.89-1.3) | 155 (57) | 1.3 (1.0-1.7) | 93 (47) | 0.86 (.64-1.2) | 70 (47) | 0.82 (.58-1.2) | 180 (54) | 1.1 (.89-1.5) | 527 (50) |
| *p for trend* |  | *0.90* |  | *0.37* |  | *0.43* |  | *0.15* |  | *0.68* |  |
| **rs1629176** |  |  |  |  |  |  |  |  |  |  |  |
| GG | 256 (27) | 1.0 | 71 (26) | 1.0 | 56 (28) | 1.0 | 44 (29) | 1.0 | 84 (25) | 1.0 | 284 (27) |
| AG | 494 (52) | 1.1 (.85-1.3) | 143 (53) | 1.1 (.79-1.5) | 100 (50) | 0.97 (.68-1.4) | 82 (55) | 1.0 (.68-1.5) | 168 (50) | 1.1 (.80-1.5) | 517 (50) |
| AA | 206 (22) | 0.93 (.72-1.2) | 57 (21) | 0.92 (.63-1.4) | 44 (22) | 0.92 (.60-1.4) | 24 (16) | 0.62 (.36-1.0) | 81 (24) | 1.1 (.78-1.6) | 243 (23) |
| AG/AA | 700 (73) | 1.0 (.83-1.2) | 200 (74) | 1.0 (.77-1.4) | 144 (72) | 0.95 (.68-1.3) | 106 (71) | 0.88 (.60-1.3) | 249 (75) | 1.1 (.82-1.4) | 760 (73) |
| *p for trend* |  | *0.62* |  | *0.73* |  | *0.71* |  | *0.10* |  | *0.59* |  |
| **rs1715385** |  |  |  |  |  |  |  |  |  |  |  |
| GG | 234 (24) | 1.0 | 66 (24) | 1.0 | 49 (25) | 1.0 | 33 (22) | 1.0 | 86 (26) | 1.0 | 269 (26) |
| AG | 483 (51) | 1.1 (.87-1.3) | 148 (55) | 1.2 (.85-1.6) | 101 (51) | 1.1 (.74-1.6) | 67 (44) | 1.1 (.70-1.7) | 166 (50) | 1.0 (.75-1.4) | 514 (49) |
| AA | 239 (25) | 1.1 (.83-1.4) | 57 (21) | 0.90 (.61-1.3) | 50 (25) | 1.1 (.70-1.7) | 51 (34) | 1.6 (.98-2.5) | 80 (24) | 0.97 (.68-1.4) | 260 (25) |
| AG/AA | 722 (76) | 1.1 (.88-1.3) | 205 (76) | 1.1 (.80-1.5) | 151 (76) | 1.1 (.75-1.5) | 118 (75) | 1.3 (.83-1.9) | 246 (74) | 1.0 (.75-1.3) | 774 (74) |
| *p for trend* |  | *0.65* |  | *0.66* |  | *0.75* |  | *0.05* |  | *0.85* |  |
| **rs6133175** |  |  |  |  |  |  |  |  |  |  |  |
| AA | 417 (44) | 1.0 | 127 (47) | 1.0 | 92 (46) | 1.0 | 53 (36) | 1.0 | 145 (44) | 1.0 | 463 (44) |
| AG | 429 (45) | 1.0 (.85-1.2) | 122 (46) | 0.96 (.72-1.3) | 86 (43) | 0.93 (.67-1.3) | 70 (47) | 1.3 (.91-1.9) | 149 (45) | 1.0 (.79-1.3) | 465 (45) |
| GG | 105 (11) | 1.0 (.75-1.4) | 19 (7.1) | 0.60 (.36-1.0) | 22 (11) | 0.99 (.59-1.6) | 26 (17) | **1.9 (1.1-3.2)** | 38 (11) | 1.0 (.69-1.6) | 115 (11) |
| AG/GG | 534 (56) | 1.0 (.86-1.2) | 141 (53) | 0.89 (.68-1.2) | 108 (54) | 0.94 (.69-1.3) | 96 (64) | 1.4 (1.0-2.1) | 187 (56) | 1.0 (.80-1.3) | 580 (56) |
| *p for trend* |  | *0.86* |  | *0.12* |  | *0.80* |  | *0.01* |  | *0.80* |  |
| **rs1715364** |  |  |  |  |  |  |  |  |  |  |  |
| TT | 275 (29) | 1.0 | 77 (28) | 1.0 | 63 (32) | 1.0 | 37 (25) | 1.0 | 98 (30) | 1.0 | 319 (30) |
| CT | 473 (49) | 1.0 (.84-1.3) | 145 (54) | 1.1 (.83-1.5) | 96 (48) | 0.91 (.64-1.3) | 68 (45) | 1.1 (.74-1.7) | 160 (48) | 0.98 (.74-1.3) | 531 (51) |
| CC | 210 (22) | 1.2 (.95-1.6) | 49 (18) | 1.0 (.69-1.5) | 41 (21) | 1.1 (0.70-1.7) | 46 (30) | **1.9 (1.2-3.1)** | 73 (22) | 1.2 (.84-1.7) | 198 (19) |
| CT/CC | 680 (71) | 1.1 (.89-1.3) | 194 (72) | 1.1 (.82-1.5) | 137 (69) | 0.95 (.69-1.3) | 114 (76) | 1.4 (.92-2.0) | 233 (70) | 1.0 (.79-1.4) | 729 (70) |
| *p for trend* |  | *0.14* |  | *0.78* |  | *0.86* |  | *6.4 x10^-3^3* |  | *0.39* |  |
| **rs1776948** |  |  |  |  |  |  |  |  |  |  |  |
| GG | 258 (27) | 1.0 | 76 (28) | 1.0 | 56 (28) | 1.0 | 35 (24) | 1.0 | 91 (28) | 1.0 | 322 (31) |
| AG | 460 (48) | 1.1 (.92-1.4) | 139 (52) | 1.2 (.84-1.6) | 90 (45) | 1.0 (.70-1.4) | 70 (47) | 1.3 (.84-2.0) | 160 (49) | 1.1 (.83-1.5) | 512 (50) |
| AA | 231 (24) | 1.4 (1.1-1.9) | 53 (20) | 1.1 (.76-1.7) | 55 (27) | **1.6 (1.1-2.4)** | 44 (30) | **2.0 (1.2-3.2)** | 78 (24) | 1.4 (.96-1.9) | 200 (19) |
| AG/AA | 691 (73) | 1.2 (1.0-1.5) | 192 (72) | 1.1 (.85-1.5) | 145 (69) | 1.2 (.83-1.6) | 114 (77) | 1.5 (1.0-2.2) | 238 (72) | 1.2 (.90-1.6) | 712 (69) |
| *p for trend* |  | *5.2E-03* |  | *0.49* |  | ***0.04*** |  | ***4.6x10^-3^*** |  | *0.08* |  |
| **rs6139587** |  |  |  |  |  |  |  |  |  |  |  |
| AA | 316 (33) | 1.0 | 81 (30) | 1.0 | 62 (33) | 1.0 | 56 (37) | 1.0 | 116 (35) | 1.0 | 350 (33) |
| AT | 480 (50) | 1.1 (.88-1.3) | 133 (49) | 1.2 (.86-1.6) | 104 (47) | 1.2 (.86-1.7) | 68 (45) | 0.84 (.57-1.2) | 174 (52) | 1.1 (.81-1.4) | 494 (47) |
| TT | 161 (17) | 0.88 (.68-1.1) | 56 (21) | 1.2 (.82-1.8) | 35 (19) | 0.97 (.62-1.5) | 27 (18) | 0.82 (.50-1.3) | 43 (13) | **0.64 (.43-.95)** | 204 (19) |
| AT/TT | 641 (67) | 1.0 (.85-1.2) | 189 (70) | 1.2 (.88-1.6) | 139 (67) | 1.1 (.82-1.6) | 95 (63) | 0.84 (.59-1.2) | 217 (65) | 0.94 (.72-1.2) | 698 (67) |
| *p for trend* |  | *0.49* |  | *0.30* |  | *0.89* |  | *0.38* |  | *0.08* |  |
