# Supplementary material for: Polymorphisms in the Estrogen Receptor 1 and Vitamin C and Matrix Metalloproteinase Gene Families Are Associated with Susceptibility to Lymphoma
Source: PLoS One. 2008 Jul 30;3(7):e2816. doi: 10.1371/journal.pone.0002816 (PMC2474696; doi:10.1371/journal.pone.0002816)
Supplement: Table S2 — Odds ratios (OR) and 95% confidence intervals (CI) for associated MMP1, 3 and 9 SNPs in the San Francisco Bay Area NHL study. (0.14 MB DOC) [file pone.0002816.s006.doc]

**Table S2. Odds ratios (OR) and 95% confidence intervals (CI) for associated *MMP1, 3 and 9*** SNPs in the San Francisco Bay Area NHL study.

| SNP & Genotype | All NHL (N=959) n(%) | OR(95% CI) | DL (N=271) n(%) | OR(95% CI) | FL (N=201) n(%) | OR(95% CI) | SLL (N=151) n(%) | OR(95% CI) | Other (N=334) n(%) | OR(95% CI) | Controls (N=1049) n(%) |
| --- | --- | --- | --- | --- | --- | --- | --- | --- | --- | --- | --- |
| MMP1 |  |  |  |  |  |  |  |  |  |  |  |
| **rs495366** |  |  |  |  |  |  |  |  |  |  |  |
| GG | 551 (58) | 1.0 | 155 (57) | 1.0 | 124 (62) | 1.0 | 81 (54) | 1.0 | 190 (57) | 1.0 | 553 (53) |
| AG | 321 (34) | 0.78 (.64-.94) | 88 (32) | 0.75 (.56-1.0) | 66 (33) | 0.71 (.51-.98) | 55 (37) | 0.90 (.63-1.3) | 111 (33) | 0.78 (.59-1.0) | 415 (40) |
| AA | 84 (8.8) | 1.1 (.78-1.5) | 28 (10) | 1.3 (.80-2.0) | 11 (5.5) | 0.63 (.33-1.2) | 13 (8.7) | 1.1 (.60-2.1) | 32 (9.6) | 1.2 (.76-1.8) | 78 (7.5) |
| AG/AA | 405 (42) | 0.83 (.69-.99) | 116 (43) | 0.84 (.64-1.1) | 77 (38) | **0.69 (.51-.94)** | 68 (46) | 0.94 (.67-1.3) | 143 (43) | 0.84 (.66-1.1) | 493 (47) |
| *p for trend* |  | *0.24* |  | *0.73* |  | ***0.02*** |  | *0.96* |  | *0.59* |  |
| **rs1144396** |  |  |  |  |  |  |  |  |  |  |  |
| AA | 259 (27) | 1.0 | 74 (27) | 1.0 | 41 (20) | 1.0 | 46 (31) | 1.0 | 98 (29) | 1.0 | 273 (26) |
| AC | 433 (45) | 0.90 (.73-1.1) | 130 (48) | 0.94 (.68-1.3) | 98 (49) | 1.3 (.86-1.9) | 62 (42) | 0.72 (.48-1.1) | 142 (43) | 0.78 (.58-1.0) | 511 (45) |
| CC | 263 (28) | 1.1 (.83-1.3) | 66 (24) | 0.93 (.64-1.3) | 62 (31) | 1.6 (1.0-2.4) | 41 (28) | 0.94 (.60-1.5) | 93 (28) | 0.99 (.71-1.4) | 263 (28) |
| AC/CC | 696 (73) | 0.95 (.78-1.2) | 196 (73) | 0.94 (.69-1.3) | 160 (80) | 1.4 (.95-2.0) | 103 (69) | 0.80 (.55-1.2) | 235 (71) | 0.85 (.65-1.1) | 774 (73) |
| *p for trend* |  | *0.66* |  | *0.69* |  | ***0.04*** |  | *0.76* |  | *0.92* |  |
| **rs529381** |  |  |  |  |  |  |  |  |  |  |  |
| GG | 531 (56) | 1.0 | 145 (54) | 1.0 | 119 (60) | 1.0 | 82 (55) | 1.0 | 184 (55) | 1.0 | 542 (52) |
| AG | 329 (34) | 0.80(.67-.97) | 93 (34) | 0.83 (.62-1.1) | 67 (34) | 0.72 (.52-1.0) | 53 (26) | 0.84 (.58-1.2) | 115 (34) | 0.81 (.62-1.1) | 419 (40) |
| AA | 96 (10) | 1.1 (.81-1.5) | 33 (12) | 1.4 (.90-2.2) | 14 (7.0) | 0.73 (.40-1.3) | 14 (9.4) | 1.0 (.56-1.9) | 35 (10) | 1.2 (.75-1.8) | 88 (8.4) |
| AG/AA | 425 (44) | 0.86 (.72-1.0) | 126 (46) | 0.93 (.71-1.2) | 81 (41) | 0.72 (.53-.99) | 67 (45) | 0.87 (.62-1.2) | 150 (44) | 0.87 (.68-1.1) | 507 (48) |
| *p for trend* |  | *0.45* |  | *0.68* |  | *0.06* |  | *0.64* |  | *0.70* |  |
| **rs1144397** |  |  |  |  |  |  |  |  |  |  |  |
| GG | 362 (38) | 1.0 | 96 (35) | 1.0 | 85 (43) | 1.0 | 57 (38) | 1.0 | 123 (37) | 1.0 | 366 (35) |
| GT | 412 (43) | 0.86 (.70-1.0) | 121 (45) | 0.95 (.70-1.3) | 87 (44) | 0.77 (.55-1.1) | 60 (40) | 0.78 (.53-1.2) | 143 (43) | 0.87 (.66-1.1) | 488 (47) |
| TT | 181 (19) | 0.96 (.74-1.2) | 54 (4.1) | 1.1 (.73-1.6) | 28 (14) | 0.63 (.40-1.0) | 32 (21) | 1.1 (.67-1.7) | 67 (20) | 1.0 (.74-1.5) | 191 (18) |
| GT/TT | 593 (62) | 0.88 (.74-1.1) | 175 (65) | 0.98 (.74-1.3) | 115 (58) | 0.73 (.53-1.0) | 92 (62) | 0.87 (.61-1.2) | 210 (63) | 0.92 (.71-1.2) | 679 (65) |
| *p for trend* |  | *0.49* |  | *0.83* |  | *0.03* |  | *0.98* |  | *0.97* |  |
| ***MMP3*** |  |  |  |  |  |  |  |  |  |  |  |
| **rs564018** |  |  |  |  |  |  |  |  |  |  |  |
| CC | 722 (75) | 1.0 | 205 (76) | 1.0 | 156 (78) | 1.0 | 111 (74) | 1.0 | 248 (74) | 1.0 | 788 (75) |
| CT | 216 (23) | 0.99 (.80-1.2) | 59 (22) | 0.96 (.69-1.3) | 42 (21) | 0.89 (.62-1.3) | 36 (24) | 1.1 (.72-1.6) | 79 (24) | 1.1 (.80-1.4) | 238 (23) |
| TT | 20 (2.1) | 1.1 (.60-2.2) | 7 (2.6) | 1.4 (.56-3.3) | 3 (1.5) | 0.76 (.22-2.6) | 4 (2.7) | 1.6 (.52-4.8) | 6 (1.8) | 1.0 (.40-2.6) | 19 (1.8) |
| CT/TT | 236 (25) | 1.0 (.82-1.2) | 66 (24) | 0.99 (.72-1.3) | 45 (22) | 0.88 (.62-1.3) | 40 (26) | 1.1 (.75-1.6) | 85 (26) | 1.1 (.80-1.4) | 257 (25) |
| *p for trend* |  | *0.90* |  | *0.90* |  | *0.48* |  | *0.48* |  | *0.72* |  |
| **rs679620** |  |  |  |  |  |  |  |  |  |  |  |
| AA | 252 (26) | 1.0 | 72 (27) | 1.0 | 38 (19) | 1.0 | 47 (32) | 1.0 | 95 (29) | 1.0 | 270 (26) |
| AG | 442 (46) | 0.93 (.75-1.2) | 132 (49) | 0.97 (.70-1.3) | 98 (49) | 1.4 (.91-2.0) | 64 (43) | 0.72 (.48-1.1) | 147 (44) | 0.82 (.61-1.1) | 509 (49) |
| GG | 260 (27) | 1.0 (.82-1.3) | 66 (24) | 0.92 (.63-1.3) | 64 (32) | **1.7 (1.1-2.6)** | 38 (26) | 0.82 (.52-1.3) | 91 (27) | 0.97 (.69-1.3) | 269 (26) |
| AG/GG | 702 (74) | 0.97 (.79-1.2) | 198 (73) | 0.95 (.70-1.3) | 162 (81) | 1.5 (1.0-2.2) | 102 (68) | 0.76 (.52-1.1) | 238 (71) | 0.87 (.66-1.1) | 778 (74) |
| *p for trend* |  | *0.75* |  | *0.67* |  | *0.02* |  | *0.38* |  | *0.82* |  |
| **rs615098** |  |  |  |  |  |  |  |  |  |  |  |
| CC | 640 (67) | 1.0 | 175 (65) | 1.0 | 142 (72) | 1.0 | 95 (63) | 1.0 | 226 (68) | 1.0 | 687 (66) |
| CA | 274 (29) | 0.91 (.75-1.1) | 85 (31) | 1.0 (.77-1.4) | 49 (25) | 0.74 (.52-1.0) | 51 (34) | 1.1 (.78-1.6) | 89 (27) | 0.83 (.63-1.1) | 324 (31) |
| AA | 38 (4.0) | 1.2 (.74-1.9) | 10 (23) | 1.1 (.55-2.3) | 6 (3.1) | 0.86 (.35-2.1) | 5 (3.3) | 1.1 (.40-2.8) | 17 (5.1) | 1.5 (.82-2.8) | 34 (3.3) |
| CA/AA | 312 (33) | 0.93 (.77-1.1) | 95 (55) | 1.0 (.79-1.4) | 55 (28) | 0.75 (.53-1.0) | 56 (37) | 1.1 (.79-1.6) | 106 (32) | 0.90 (.69-1.2) | 358 (34) |
| *p for trend* |  | *0.73* |  | *0.72* |  | *0.13* |  | *0.58* |  | *0.87* |  |
| MMP9 |  |  |  |  |  |  |  |  |  |  |  |
| **rs6094237** |  |  |  |  |  |  |  |  |  |  |  |
| AA | 341 (36) | 1.0 | 102 (38) | 1.0 | 66 (33) | 1.0 | 48 (32) | 1.0 | 123 (37) | 1.0 | 360 (35) |
| AT | 438 (46) | 0.91 (.75-1.1) | 129 (48) | 0.89 (.67-1.2) | 105 (52) | 1.1 (.82-1.6) | 69 (46) | 1.0 (.67-1.5) | 135 (41) | 0.77 (.58-1.0) | 505 (49) |
| TT | 175 (18) | 1.1 (.82-1.4) | 38 (14) | 0.77 (.51-1.2) | 30 (15) | 0.96 (.60-1.5) | 32 (21) | 1.3 (.83-2.2) | 75 (23) | 1.3 (.90-1.8) | 173 (17) |
| AT/TT | 613 (64) | 0.95 (.79-1.1) | 167 (62) | 0.86 (.65-1.1) | 135 (67) | 1.1 (.79-1.5) | 101 (68) | 1.1 (.75-1.6) | 210 (63) | 0.89 (.69-1.2) | 678 (65) |
| *p for trend* |  | *0.89* |  | *0.21* |  | *0.94* |  | *0.29* |  | *0.47* |  |
| **rs4810482** |  |  |  |  |  |  |  |  |  |  |  |
| TT | 379 (40) | 1.0 | 120 (44) | 1.0 | 69 (35) | 1.0 | 56 (37) | 1.0 | 132 (40) | 1.0 | 424 (41) |
| CT | 427 (45) | 0.97 (.80-1.2) | 120 (44) | 0.86 (.65-1.1) | 104 (52) | 1.3 (.94-1.8) | 65 (43) | 0.99 (.68-1.4) | 138 (42) | 0.90 (.68-1.2) | 490 (47) |
| CC | 149 (16) | 1.3 (.95-1.6) | 31 (11) | 0.83 (.53-1.3) | 26 (13) | 1.2 (.74-2.0) | 30 (20) | 1.7 (1.0-2.7) | 62 (19) | 1.5 (1.0-2.1) | 133 (13) |
| CT/CC | 576 (60) | 1.0 (.86-1.2) | 151 (56) | 0.85 (.65-1.1) | 130 (65) | 1.3 (.94-1.8) | 95 (63) | 1.1 (.80-1.6) | 200 (60) | 1.0 (.79-1.3) | 623 (60) |
| *p for trend* |  | *0.24* |  | *0.27* |  | *0.23* |  | *0.09* |  | *0.13* |  |
| **rs3918241** |  |  |  |  |  |  |  |  |  |  |  |
| TT | 656 (69) | 1.0 | 199 (74) | 1.0 | 135 (68) | 1.0 | 95 (63) | 1.0 | 225 (68) | 1.0 | 761 (73) |
| AT | 271 (28) | 1.2 (.99-1.5) | 64 (24) | 0.94 (.69-1.3) | 62 (31) | 1.3 (.95-1.9) | 50 (33) | 1.6 (1.1-2.3) | 95 (29) | 1.2 (.94-1.6) | 260 (25) |
| AA | 30 (3.1) | 1.7 (.95-3.0) | 7 (2.6) | 1.3 (.54-3.1) | 4 (2.0) | 1.1 (.36-3.1) | 6 (4.0) | 2.4 (.93-6.1) | 13 (3.9) | 2.2 (1.0-4.4) | 21 (2.0) |
| AT/AA | 301 (31) | 1.2 (1.0-1.5) | 71 (26) | 0.97 (.71-1.3) | 66 (33) | 1.3 (.95-1.8) | 56 (37) | **1.6 (1.1-2.3)** | 108 (32) | 1.3 (1.0-1.7) | 281 27) |
| *p for trend* |  | ***0.01*** |  | *0.99* |  | *0.15* |  | ***4.8x10^-3^*** |  | ***0.02*** |  |
| **rs17576** |  |  |  |  |  |  |  |  |  |  |  |
| AA | 378 (40) | 1.0 | 115 (43) | 1.0 | 73 (37) | 1.0 | 54 (36) | 1.0 | 134 (41) | 1.0 | 424 (41) |
| AG | 421 (45) | 0.98 (.81-1.2) | 120 (45) | 0.92 (.69-1.2) | 101 (51) | 1.2 (.88-1.7) | 65 (44) | 1.1 (.72-1.5) | 135 (41) | 0.88 (.67-1.2) | 481 (46) |
| GG | 145 (15) | 1.2 (.95-1.6) | 30 (11) | 0.86 (.55-1.3) | 24 (12) | 1.1 (.65-1.8) | 30 (20) | **1.8 (1.1-3.0)** | 61 (18) | 1.5 (1.0-2.1) | 130 (13) |
| AG/GG | 566 (60) | 1.0 (.87-1.2) | 150 (57) | 0.90 (.69-1.2) | 125 (63) | 1.2 (.87-1.6) | 95 (64) | 1.2 (.85-1.7) | 196 (59) | 1.0 (.78-1.3) | 611 (59) |
| *p for trend* |  | *0.24* |  | *0.43* |  | *0.49* |  | ***0.04*** |  | *0.15* |  |
| **rs2274756** |  |  |  |  |  |  |  |  |  |  |  |
| GG | 658 (69) | 1.0 | 199 (74) | 1.0 | 137 (68) | 1.0 | 95 (63) | 1.0 | 225 (68) | 1.0 | 763 (73) |
| AG | 268 (28) | 1.2 (.99-1.5) | 64 (24) | 0.94 (.69-1.3) | 60 (30) | 1.3 (.95-1.9) | 50 (33) | **1.6 (1.1-2.3)** | 94 (28) | 1.2 (.94-1.6) | 258 (25) |
| AA | 30 (3.1) | 1.7 (.95-3.0) | 7 (2.6) | 1.3 (.54-3.1) | 4 (2.0) | 1.1 (.36-3.1) | 6 (4.0) | 2.4 (.93-6.1) | 13 (3.9) | 2.2 (1.0-4.4) | 20 (1.9) |
| AG/AA | 298 (31) | 1.2 (1.0-1.5) | 71 (26) | 0.97 (.71-1.3) | 64 (32) | 1.3 (.95-1.8) | 56 (37) | 1.6 (1.1-2.3) | 107 (32) | 1.3 (1.0-1.7) | 278 (27) |
| *p for trend* |  | *0.01* |  | *0.99* |  | *0.15* |  | ***4.8x10^-3^*** |  | ***0.02*** |  |
