# Supplementary material for: Polymorphisms in the Estrogen Receptor 1 and Vitamin C and Matrix Metalloproteinase Gene Families Are Associated with Susceptibility to Lymphoma
Source: PLoS One. 2008 Jul 30;3(7):e2816. doi: 10.1371/journal.pone.0002816 (PMC2474696; doi:10.1371/journal.pone.0002816)
Supplement: Table S3 — Odds ratios (OR) and 95% confidence intervals (CI) for associated CYP19A1 SNPs in the San Francisco Bay Area NHL study. (0.16 MB DOC) [file pone.0002816.s007.doc]

**Table S3. Odds ratios (OR) and 95% confidence intervals (CI) for associated *CYP19A1* SNPs in the San Francisco Bay Area NHL study.**

| SNP & Genotype | All NHL (N=959) n(%) | OR(95% CI) | DL (N=271) n(%) | OR(95% CI) | FL (N=201) n(%) | OR(95% CI) | SLL (N=151) n(%) | OR(95% CI) | Other (N=334) n(%) | OR(95% CI) | Controls (N=1049) n(%) |
| --- | --- | --- | --- | --- | --- | --- | --- | --- | --- | --- | --- |
| **rs10046** |  |  |  |  |  |  |  |  |  |  |  |
| TT | 265 (28) | 1.0 | 72 (27) | 1.0 | 60 (30) | 1.0 | 54 (36) | 1.0 | 79 (24) | 1.0 | 283 (27) |
| CT | 471 (49) | 0.98 (.79-1.2) | 137 (51) | 1.1 (.76-1.4) | 96 (48) | 0.88 (.62-1.3) | 66 (44) | **0.66 (.45-.98)** | 172 (52) | 1.2 (.88-1.6) | 513 (49) |
| CC | 219 (23) | 0.93 (.73-1.2) | 61 (23) | 0.96 (.66-1.4) | 44 (22) | 0.83 (.54-1.3) | 30 (20) | **0.61 (.38-.99)** | 82 (25) | 1.2 (.82-1.7) | 251 (24) |
| CT/CC | 690 (72) | 0.96 (.79-1.2) | 198 (73) | 1.0 (.75-1.4) | 140 (70) | 0.87 (.62-1.2) | 96 (64) | **0.65 (.45-.93)** | 254 (76) | 1.2 (.89-1.6) | 764 (73) |
| *p for trend* |  | *0.59* |  | *0.85* |  | *0.38* |  | ***0.03*** |  | *0.40* |  |
| **rs2289105** |  |  |  |  |  |  |  |  |  |  |  |
| CC | 267 (28) | 1.0 | 72 (27) | 1.0 | 60 (30) | 1.0 | 54 (36) | 1.0 | 81 (24) | 1.0 | 286 (27) |
| CT | 476 (50) | 0.99 (.80-1.2) | 138 (51) | 1.1 (.77-1.5) | 98 (49) | 0.91 (.64-1.3) | 67 (44) | 0.68 (.46-1.0) | 173 (52) | 1.2 (.87-1.6) | 514 (49) |
| TT | 216 (23) | 0.94 (.73-1.2) | 61 (23) | 0.99 (.67-1.4) | 43 (21) | 0.84 (.54-1.3) | 30 (20) | 0.63 (.39-1.0) | 80 (24) | 1.1 (.80-1.6) | 247 (24) |
| CT/TT | 692 (72) | 0.97 (.80-1.2) | 199 (73) | 1.0 (.77-1.4) | 141 (70) | 0.88 (.63-1.2) | 97 (64) | **0.66 (.46-.95)** | 253 (76) | 1.2 (.88-1.5) | 761 (73) |
| *p for trend* |  | *0.63* |  | *0.97* |  | *0.41* |  | ***0.04*** |  | *0.46* |  |
| **rs2899472** |  |  |  |  |  |  |  |  |  |  |  |
| CC | 498 (52) | 1.0 | 146 (54) | 1.0 | 100 (50) | 1.0 | 66 (44) | 1.0 | 184 (55) | 1.0 | 597 (57) |
| AC | 390 (41) | 1.2 (1.0-1.5) | 112 (41) | 1.2 (.92-1.6) | 80 (40) | 1.3 (.92-1.8) | 67 (44) | **1.6 (1.1-2.3)** | 131 (39) | 1.1 (.87-1.5) | 378 (36) |
| AA | 71 (7.4) | 1.1 (.81-1.6) | 13 (4.8) | 0.72 (.39-1.3) | 21 (10) | 1.7 (1.0-2.9) | 18 (12) | **2.2 (1.3-4.0)** | 19 (5.7) | 0.84 (.49-1.4) | 74 (7.1) |
| AC/AA | 461 (48) | 1.2 (1.0-1.5) | 125 (46) | 1.1 (.87-1.5) | 101 (50) | 1.3 (.99-1.8) | 85 (56) | **1.7 (1.2-2.4)** | 150 (45) | 1.1 (.85-1.4) | 452 (43) |
| *p for trend* |  | *0.06* |  | *0.84* |  | *0.03* |  | ***1.2x10^-3^*** |  | *0.87* |  |
| **rs9944225** |  |  |  |  |  |  |  |  |  |  |  |
| CC | 786 (82) | 1.0 | 218 (80) | 1.0 | 163 (82) | 1.0 | 138 (91) | 1.0 | 266 (80) | 1.0 | 867 (83) |
| AC | 166 (17) | 1.0 (.81-1.3) | 51 (19) | 1.1 (.81-1.6) | 36 (18) | 1.1 (.73-1.6) | 12 (8) | 0.42 (.23-.77) | 66 (20) | 1.2 (.88-1.6) | 177 (17) |
| AA | 6 (.63) | 1.4 (.42-4.5) | 2 (.74) | 1.6 (.31-8.3) | 1 (.50) | 1.0 (.12-9.1) | 1 (.66) | 1.4 (.16-12) | 2 (.60) | 1.3 (.25-6.8) | 5 (.48) |
| AC/AA | 172 (18) | 1.0 (.82-1.3) | 53 (20) | 1.2 (.82-1.6) | 37 (19) | 1.1 (.74-1.6) | 13 (9.0) | **0.44 (.24-.79)** | 68 (20) | 1.2 (.88-1.6) | 182 (17) |
| *p for trend* |  | *0.70* |  | *0.37* |  | *0.68* |  | ***0.01*** |  | *0.24* |  |
|  |  |  |  |  |  |  |  |  |  |  |  |
| **rs4775936** |  |  |  |  |  |  |  |  |  |  |  |
| TT | 263 (27) | 1.0 | 70 (26) | 1.0 | 57 (29) | 1.0 | 35 (23) | 1.0 | 99 (30) | 1.0 | 304 (29) |
| CT | 487 (51) | 1.1 (.90-1.4) | 138 (51) | 1.2 (.85-1.6) | 98 (49) | 1.0 (.71-1.4) | 75 (50) | 1.3 (.85-2.0) | 176 (53) | 1.1 (.80-1.4) | 512 (49) |
| CC | 208 (22) | 1.0 (.81-1.3) | 63 (23) | 1.2 (.81-1.7) | 45 (23) | 1.0 (.68-1.6) | 41 (27) | 1.6 (.97-2.6) | 59 (18) | 0.79 (.55-1.1) | 232 (22) |
| CT/CC | 695 (73) | 1.1 (.89-1.3) | 201 (74) | 1.2 (.87-1.6) | 143 (72) | 1.0 (.73-1.4) | 116 (77) | 1.4 (.93-2.1) | 235 (70) | 0.98 (.75-1.3) | 744 (71) |
| *p for trend* |  | *0.72* |  | *0.37* |  | *0.87* |  | *0.07* |  | *0.27* |  |
| **rs2008691** |  |  |  |  |  |  |  |  |  |  |  |
| AA | 700 (73) | 1.0 | 197 (73) | 1.0 | 149 (74) | 1.0 | 114 (76) | 1.0 | 239 (72) | 1.0 | 784 (75) |
| AG | 236 (25) | 1.1 (.86-1.3) | 70 (26) | 1.1 (.82-1.5) | 48 (24) | 1.0 (.71-1.5) | 33 (22) | 0.89 (.58-1.3) | 84 (25) | 1.1 (.82-1.5) | 205 (24) |
| GG | 23 (2.4) | 2.0 (.99-3.9) | 4 (1.5) | 1.3 (.40-3.9) | 4 (2.0) | 1.7 (.56-5.4) | 4 (2.7) | 2.0 (.63-6.3) | 11 (3.3) | **2.7 (1.2-6.2)** | 13 (1.2) |
| AG/GG | 259 (27) | 1.1 (.90-1.3) | 74 (27) | 1.1 (.83-1.5) | 52 (26) | 1.1 (.74-1.5) | 37 (25) | 0.94 (.63-1.4) | 95 (28) | 1.2 (.89-1.5) | 263 (25) |
| *p for trend* |  | *0.17* |  | *0.45* |  | *0.62* |  | *0.97* |  | *0.09* |  |
| **rs4774584** |  |  |  |  |  |  |  |  |  |  |  |
| GG | 287 (30) | 1.0 | 75 (28) | 1.0 | 69 (34) | 1.0 | 35 (23) | 1.0 | 106 (32) | 1.0 | 326 (31) |
| AG | 478 (50) | 1.0 (.85-1.3) | 137 (51) | 1.1 (.83-1.6) | 89 (44) | 0.80 (.57-1.1) | 77 (51) | 1.4 (.90-2.1) | 175 (53) | 1.0 (.78-1.4) | 521 (50) |
| AA | 192 (20) | 1.1 (.84-1.4) | 59 (22) | 1.3 (.87-1.9) | 43 (21) | 1.0 (.67-1.6) | 39 (26) | **1.8 (1.1-3.0)** | 51 (15) | 0.78 (.54-1.1) | 200 (19) |
| AG/AA | 670 (70) | 1.1 (.87-1.3) | 196 (72) | 1.2 (.88-1.6) | 132 (66) | 0.86 (.63-1.2) | 116 (77) | 1.5 (1.0-2.2) | 226 (68) | 0.97 (.74-1.3) | 721 (69) |
| *p for trend* |  | *0.52* |  | *0.21* |  | *0.87* |  | ***0.02*** |  | *0.30* |  |
| **rs7181886** |  |  |  |  |  |  |  |  |  |  |  |
| CC | 887 (93) | 1.0 | 249 (92) | 1.0 | 188 (94) | 1.0 | 143 (95) | 1.0 | 306 (92) | 1.0 | 973 (93) |
| CG | 67 (7.0) | 1.1 (.74-1.5) | 22 (8.1) | 1.2 (.74-2.0) | 13 (6.5) | 0.97 (.52-1.8) | 7 (4.6) | 0.68 (.31-1.5) | 24 (7.2) | 1.1 (.68-1.8) | 70 (6.7) |
| GG | 3 (.31) | 0.54 (.14-2.2) | 0 |  | 0 |  | 1 (.66) | 1.1 (.13-9.5) | 2 (.60) | 1.0 (.20-5.1) | 6 (.57) |
| CG/GG | 70 (7.3) | 1.0 (.72-1.4) | 22 (8.1) | 1.1 (.69-1.9) | 13 (6.5) | 0.89 (.49-1.6) | 8 (5.3) | 0.72 (.34-1.5) | 26 (7.8) | 1.1 (.69-1.7) | 76 7.2) |
| *p for trend* |  | *0.88* |  | *0.88* |  | *0.56* |  | *0.46* |  | *0.74* |  |
|  |  |  |  |  |  |  |  |  |  |  |  |
| **rs936306** |  |  |  |  |  |  |  |  |  |  |  |
| CC | 691 (72) | 1.0 | 196 (73) | 1.0 | 146 (73) | 1.0 | 109 (72) | 1.0 | 239 (72) | 1.0 | 782 (75) |
| CT | 241 (25) | 1.1 (.89-1.3) | 70 (26) | 1.1 (.82-1.5) | 50 (25) | 1.1 (.76-1.5) | 37 (25) | 1.0 (.70-1.6) | 83 (25) | 1.1 (.81-1.4) | 249 (24) |
| TT | 24 (2.5) | 1.9 (.99-3.8) | 4 (1.5) | 1.2 (.38-3.6) | 4 (2.0) | 1.6 (.52-4.9) | 5 (3.3) | 2.5 (.86-7.0) | 11 (3.3) | **2.6 (1.2-5.8)** | 14 (1.3) |
| CT/TT | 265 (28) | 1.1 (.93-1.4) | 74 (27) | 1.1 (.83-1.5) | 54 (27) | 1.1 (.79-1.6) | 42 (28) | 1.1 (.76-1.6) | 94 (28) | 1.2 (.88-1.5) | 263 (25) |
| *p for trend* |  | *0.10* |  | *0.46* |  | *0.46* |  | *0.33* |  | *0.11* |  |
| **rs1004982** |  |  |  |  |  |  |  |  |  |  |  |
| AA | 350 (37) | 1.0 | 101 (37) | 1.0 | 62 (31) | 1.0 | 64 (42) | 1.0 | 122 (37) | 1.0 | 392 (37) |
| AG | 478 (50) | 1.1 (.90-1.3) | 134 (49) | 1.0 (.78-1.4) | 105 (53) | 1.3 (.96-1.9) | 71 (47) | 0.87 (.60-1.3) | 167 (50) | 1.1 (.83-1.4) | 495 (47) |
| GG | 129 (13) | 0.91 (.69-1.2) | 36 (13) | 0.88 (.57-1.3) | 33 (17) | 1.3 (.83-2.1) | 16 (11) | 0.60 (.34-1.1) | 44 (13) | 0.88 (.60-1.3) | 159 (15) |
| AG/GG | 607 (63) | 1.0 (.87-1.3) | 170 (63) | 1.0 (.76-1.3) | 138 (69) | 1.3 (.97-1.9) | 87 (58) | 0.80 (.57-1.1) | 211 (63) | 1.0 (.80-1.3) | 654 (63) |
| *p for trend* |  | *0.80* |  | *0.70* |  | *0.14* |  | *0.09* |  | *0.77* |  |
| **rs2470146** |  |  |  |  |  |  |  |  |  |  |  |
| TT | 863 (90) | 1.0 | 233 (86) | 1.0 | 180 (91) | 1.0 | 135 (89) | 1.0 | 313 (94) | 1.0 | 949 (91) |
| CT | 90 (9.4) | 1.0 (.76-1.4) | 37 (14) | 1.6 (1.0-2.3) | 17 (8.6) | 0.91 (.53-1.6) | 16 (11) | 1.2 (.68-2.1) | 20 (6.0) | 0.64 (.39-1.0) | 97 (9.3) |
| CC | 1 (.10) |  | 0 |  | 0 |  | 0 |  | 1 (.30) |  | 0 |
| CT/CC | 91 (9.5) | 1.0 (.77-1.4) | 37 (14) | 1.6 (1.0-2.3) | 17 (8.6) | 0.91 (.53-1.6) | 16 (11) | 1.2 (.68-2.1) | 21 (6.3) | 0.67 (.41-1.1) | 97 (9.3) |
| *p for trend* |  | *0.75* |  | ***0.03*** |  | *0.74* |  | *0.54* |  | *0.16* |  |
| **rs2470144** |  |  |  |  |  |  |  |  |  |  |  |
| GG | 244 (26) | 1.0 | 67 (25) | 1.0 | 44 (22) | 1.0 | 44 (29) | 1.0 | 88 (26) | 1.0 | 287 (27) |
| AG | 503 (53) | 1.1 (.93-1.4) | 139 (51) | 1.1 (.83-1.6) | 104 (52) | 1.3 (.90-1.9) | 81 (54) | 1.0 (.68-1.5) | 178 (53) | 1.1 (.84-1.5) | 518 (50) |
| AA | 210 (22) | 1.0 (.81-1.3) | 64 (24) | 1.1 (.78-1.7) | 53 (26) | 1.4 (.94-2.2) | 25 (17) | 0.68 (.40-1.1) | 68 (20) | 0.93 (.65-1.3) | 239 (23) |
| AG/AA | 713 (75) | 1.1 (.91-1.4) | 203 (75) | 1.1 (.84-1.6) | 157 (78) | 1.4 (.95-2.0) | 106 (71) | 0.90 (.62-1.3) | 246 (74) | 1.1 (.80-1.4) | 757 (73) |
| *p for trend* |  | *0.72* |  | *0.48* |  | *0.10* |  | *0.18* |  | *0.77* |  |
| **rs1870049** |  |  |  |  |  |  |  |  |  |  |  |
| TT | 719 (75) | 1.0 | 216 (80) | 1.0 | 134 (67) | 1.0 | 108 (72) | 1.0 | 260 (78) | 1.0 | 810 (77) |
| CT | 223 (23) | 1.1 (.92-1.4) | 53 (20) | 0.89 (.64-1.2) | 59 (30) | **1.6 (1.1-2.3)** | 40 (26) | 1.3 (.91-2.0) | 70 (21) | 0.98 (.72-1.3) | 222 (21) |
| CC | 16 (1.7) | 1.3 (.64-2.7) | 2 (.74) | 0.55 (.12-2.4) | 7 (3.5) | **3.0 (1.2-7.5)** | 3 (2.0) | 1.7 (.48-6.1) | 4 (2.1) | 0.92 (.30-2.8) | 14 (1.3) |
| CT/CC | 239 (25) | 1.1 (.93-1.4) | 55 (20) | 0.87 (.63-1.2) | 66 (33) | **1.7 (1.2-2.3)** | 43 (28) | 1.4 (.93-2.0) | 74 (22) | 0.98 (.73-1.3) | 236 (23) |
| *p for trend* |  | *0.17* |  | *0.35* |  | ***8.0E-04*** |  | *0.10* |  | *0.85* |  |
| **rs752760** |  |  |  |  |  |  |  |  |  |  |  |
| TT | 300 (32) | 1.0 | 84 (31) | 1.0 | 56 (28) | 1.0 | 50 (33) | 1.0 | 109 (33) | 1.0 | 335 (32) |
| CT | 480 (51) | 1.1 (.88-1.3) | 131 (49) | 1.0 (.76-1.4) | 99 (50) | 1.2 (.83-1.7) | 82 (55) | 1.1 (.87-1.6) | 167 (51) | 1.0 (.77-1.3) | 503 (49) |
| CC | 167 (18) | 0.94 (.73-1.2) | 52 (19) | 1.0 (.71-1.5) | 43 (22) | 1.3 (.84-2.0) | 18 (12) | 0.60 (.34-1.1) | 54 (16) | 0.84 (.58-1.2) | 199 (19) |
| CT/CC | 647 (68) | 1.0 (.86-1.2) | 183 (69) | 1.0 (.78-1.4) | 142 (72) | 1.2 (.87-1.7) | 100 (67) | 0.93 (.65-1.3) | 221 (67) | 0.97 (.74-1.3) | 702 (68) |
| *p for trend* |  | *0.79* |  | *0.83* |  | *0.23* |  | *0.16* |  | *0.43* |  |
