# Supplementary material for: Polymorphisms in the Estrogen Receptor 1 and Vitamin C and Matrix Metalloproteinase Gene Families Are Associated with Susceptibility to Lymphoma
Source: PLoS One. 2008 Jul 30;3(7):e2816. doi: 10.1371/journal.pone.0002816 (PMC2474696; doi:10.1371/journal.pone.0002816)
Supplement: Table S4 — Odds ratios (OR) and 95% confidence intervals (CI) for associated ESR1 SNPs in the San Francisco Bay Area NHL study. (0.12 MB DOC) [file pone.0002816.s008.doc]

**Table S4. Odds ratios (OR) and 95% confidence intervals (CI) for associated *ESR1* SNPs in the San Francisco Bay Area NHL study.**

| SNP & genotype | All NHL (N=959) n(%) | OR(95% CI) | DL (N=271) n(%) | OR(95% CI) | FL (N=201) n(%) | OR(95% CI) | SLL (N=151) n(%) | OR(95% CI) | Other NHL (N=334) n(%) | OR(95% CI) | Controls (N=1049) n(%) |
| --- | --- | --- | --- | --- | --- | --- | --- | --- | --- | --- | --- |
| **rs2881766** |  |  |  |  |  |  |  |  |  |  |  |
| TT | 633 (66) | 1.0 | 170 (63) | 1.0 | 134 (67) | 1.0 | 98 (65) | 1.0 | 229 (69) | 1.0 | 703 (67) |
| CT | 291 (30) | 1.1 (.90-1.3) | 91 (34) | 1.3 (.96-1.7) | 58 (29) | 1.0 (.73-1.4) | 51 (34) | 1.3 (.87-1.8) | 91 (27) | 0.94 (.71-1.2) | 296 (28) |
| CC | 35 (3.7) | 0.81 (.52-1.3) | 10 (4) | 0.87 (.43-1.8) | 9 (4.9) | 1.0 (.48-2.1) | 2 (1.3) | 0.30 (.07-1.2) | 14 (4.2) | 0.90 (.49-1.7) | 48 (4.6) |
| CT/CC | 326 (34) | 1.1 (.88-1.3) | 101 (37) | 1.2 (.92-1.6) | 67 (33) | 1.0 (.74-1.4) | 53 (35) |  | 105 (31) | 0.94 (.72-1.2) | 344 (33) |
| *p for trend* |  | *0.91* |  | *0.35* |  | *0.95* |  | *0.87* |  | *0.62* |  |
| **rs488133** |  |  |  |  |  |  |  |  |  |  |  |
| TT | 456 (48) | 1.0 | 130 (48) | 1.0 | 99 (51) | 1.0 | 69 (46) | 1.0 | 157 (47) | 1.0 | 472 (45) |
| CT | 402 (42) | 0.94 (.78-1.1) | 113 (42) | 0.93 (.70-1.2) | 82 (42) | 0.88 (.64-1.2) | 68 (45) | 1.1 (.75-1.5) | 139 (42) | 0.95 (.73-1.2) | 441 (42) |
| CC | 94 (10) | 0.74 (.55-.99) | 27 (10) | 0.74 (.47-1.2) | 14 (7.2) | **0.51 (.28-.93)** | 14 (9.3) | 0.73 (.40-1.3) | 38 (11) | 0.86 (.58-1.3) | 131 (13) |
| CT/CC | 496 (52) | 0.90 (.75-1.1) | 140 (52) | 0.89 (.68-1.2) | 96 (49) | 0.79 (.58-1.1) | 82 (54) | 0.99 (.70-1.4) | 177 (53) | 0.93 (.72-1.2) | 572 (55) |
| *p for trend* |  | *0.07* |  | *0.22* |  | ***0.04*** |  | *0.56* |  | *0.46* |  |
| **rs532010** |  |  |  |  |  |  |  |  |  |  |  |
| CC | 321 (34) | 1.0 | 88 (33) | 1.0 | 59 (30) | 1.0 | 54 (36) | 1.0 | 119 (36) | 1.0 | 409 (39) |
| AC | 467 (49) | 1.3 (1.0-1.5) | 136 (51) | 1.3 (.99-1.8) | 102 (51) | **1.5 (1.1-2.1)** | 71 (47) | 1.1 (.76-1.6) | 158 (48) | 1.1 (.87-1.5) | 473 (45) |
| AA | 163 (17) | 1.3 (.98-1.7) | 43 (16) | 1.2 (.83-1.9) | 38 (19) | **1.7 (1.1-2.6)** | 26 (17) | 1.2 (.70-1.9) | 55 (17) | 1.2 (.81-1.7) | 162 (16) |
| AC/AA | 630 (66) | 1.3 (1.0-1.5) | 179 (67) | 1.3 (.99-1.7) | 140 (70) | **1.5 (1.1-2.1)** | 97 (64) | 1.1 (.78-1.6) | 213 (64) | 1.1 (.89-1.5) | 635 (61) |
| *p for trend* |  | *0.03* |  | *0.15* |  | ***1.2 x10^-2^*** |  | *0.51* |  | *0.33* |  |
| **rs827423** |  |  |  |  |  |  |  |  |  |  |  |
| CC | 233 (24) | 1.0 | 69 (26) | 1.0 | 38 (19) | 1.0 | 39 (26) | 1.0 | 86 (26) | 1.0 | 287 (27) |
| AC | 484 (51) | 1.2 (.94-1.4) | 138(51) | 1.1 (.81-1.5) | 111 (55) | **1.6 (1.1-2.4)** | 73 (48) | 1.0 (.67-1.5) | 162 (49) | 1.0 (.78-1.4) | 515 (49) |
| AA | 241 (25) | 1.2 (.94-1.5) | 63 (23) | 1.1 (.74-1.6) | 52 (26) | **1.6 (1.0-2.5)** | 39 (26) | 1.1 (.70-1.8) | 86 (26) | 1.2 (.83-1.7) | 246 (23) |
| AC/AA | 725 (76) | 1.2 (.96-1.4) | 201 (74) | 1.1 (.82-1.5) | 163 (81) | **1.6 (1.1-2.4)** | 112 (74) | 1.1 (.71-1.6) | 248 (74) | 1.1 (.82-1.4) | 761 (73) |
| *p for trend* |  | *0.13* |  | *68* |  | ***9.6 x10^-3^*** |  | *0.64* |  | *0.38* |  |
| **rs1709182** |  |  |  |  |  |  |  |  |  |  |  |
| CC | 349 (36) | 1.0 | 107 (40) | 1.0 | 70 (35) | 1.0 | 49 (32) | 1.0 | 122 (37) | 1.0 | 411 (39) |
| CT | 465 (49) | 1.1 (.93-1.4) | 126 (47) | 1.0 (.75-1.3) | 97 (48) | 1.2 (.84-1.6) | 78 (52) | 1.4 (.93-2.0) | 164 (49) | 1.1 (.87-1.5) | 485 (46) |
| TT | 144 (15) | 1.1 (.87-1.5) | 37 (14) | 0.96 (.63-1.5) | 34 (17) | 1.4 (.86-2.1) | 24 (16) | 1.3 (.79-2.3) | 48 (14) | 1.1 (.74-1.6) | 148 (14) |
| CT/TT | 609 (64) | 1.1 (.94-1.4) | 163 (60) | 0.99 (.75-1.3) | 131 (65) | 1.2 (.89-1.7) | 102 (68) | 1.4 (.94-1.9) | 212 (63) | 1.1 (.88-1.5) | 633 (61) |
| *p for trend* |  | *0.22* |  | *0.88* |  | *0.17* |  | *0.17* |  | *0.49* |  |
| **rs1913474** |  |  |  |  |  |  |  |  |  |  |  |
| AA | 601 (63) | 1.0 | 170 (63) | 1.0 | 133 (66) | 1.0 | 97 (64) | 1.0 | 201 (60) | 1.0 | 622 (59) |
| AG | 313 (33) | 0.88 (.73-1.1) | 85 (31) | 0.84 (.63-1.1) | 62 (31) | 0.79 (.57-1.1) | 48 (32) | 0.83 (.57-1.2) | 116 (35) | 0.97 (.74-1.3) | 368 (35) |
| GG | 45 (4.7) | 0.80 (.54-1.2) | 16 (5.9) | 1.0 (.56-1.8) | 6 (3.0) | 0.47 (.20-1.1) | 6 (4.0) | 0.68 (.29-1.6) | 17 (5.1) | 0.91 (.52-1.6) | 58 (5.5) |
| AG/GG | 358 (37) | 0.87 (.73-1.0) | 101 (37) | 0.86 (.66-1.1) | 68 (34) | 0.75 (.54-1.0) | 54 (36) | 0.81 (.57-1.2) | 133 (40) | 0.96 (.75-1.2) | 426 (41) |
| *p for trend* |  | *0.11* |  | *0.44* |  | ***0.04*** |  | *0.22* |  | *0.72* |  |
| **rs3020314** |  |  |  |  |  |  |  |  |  |  |  |
| TT | 435 (46) | 1.0 | 122 (45) | 1.0 | 112 (56) | 1.0 | 62 (41) | 1.0 | 138 (42) | 1.0 | 450 (43) |
| TC | 423 (44) | 0.93 (.78-1.1) | 115 (43) | 0.90 (.68-1.2) | 76 (38) | **0.64 (.46-.88)** | 68 (45) | 1.1 (.74-1.5) | 163 (49) | 1.1 (.88-1.5) | 472 (45) |
| CC | 98 (10) | 0.83 (.62-1.1) | 33 (12) | 0.99 (.64-1.5) | 13 (6) | **0.42 (.23-.77)** | 21 (14) | 1.3 (.75-2.2) | 31 (9.3) | 0.83 (.54-1.3) | 123 (12) |
| AG/AA | 521 (55) | 0.91 (.76-1.1) | 148 (55) | 0.92 (.70-1.2) | 89 (44) | **0.59 (.44-.81)** | 89 (59) | 1.1 (.79-1.6) | 194 (58) | 1.1 (.84-1.4) | 595 (57) |
| *p for trend* |  | *0.21* |  | *0.72* |  | ***4.0 x10^-4^*** |  | *0.39* |  | *0.90* |  |
| **rs722208** |  |  |  |  |  |  |  |  |  |  |  |
| AA | 500 (52) | 1.0 | 142 (53) | 1.0 | 109 (55) | 1.0 | 75 (50) | 1.0 | 172 (52) | 1.0 | 550 (52) |
| AG | 376 (39) | 0.97 (.81-1.2) | 107 (40) | 0.98 (.74-1.3) | 75 (38) | 0.89 (.65-1.2) | 62 (41) | 1.1 (.74-1.5) | 132 (40) | 1.0 (.77-1.3) | 424 (40) |
| GG | 80 (8.4) | 1.2 (.85-1.7) | 21 (7.8) | 1.1 (.65-1.8) | 16 (8.0) | 1.1 (.61-1.9) | 14 (9.3) | 1.4 (.76-2.6) | 29 (8.7) | 1.3 (.80-2.0) | 74 (7.1) |
| AG/GG | 456 (48) | 1.0 (.84-1.2) | 128 (47) | 1.0 (.76-1.3) | 91 (46) | 0.92 (.68-1.2) | 76 (50) | 1.1 (.79-1.6) | 161 (48) | 1.0 (.81-1.3) | 498 (48) |
| *p for trend* |  | *0.60* |  | *0.89* |  | *0.81* |  | *0.36* |  | *0.51* |  |
| **rs3020411** |  |  |  |  |  |  |  |  |  |  |  |
| CC | 431 (45) | 1.0 | 126 (47) | 1.0 | 94 (47) | 1.0 | 65 (43) | 1.0 | 144 (43) | 1.0 | 473 (45) |
| CT | 410 (43) | 0.94 (.78-1.1) | 116 (43) | 0.91 (.69-1.2) | 79 (40) | 0.83 (.60-1.1) | 66 (44) | 0.99 (.69-1.4) | 149 (45) | 1.0 (.79-1.3) | 477 (46) |
| TT | 116 (12) | 1.3 (.98-1.8) | 28 (10) | 1.1 (.68-1.7) | 27 (14) | 1.4 (.87-2.3) | 20 (13) | 1.5 (.88-2.6) | 41 (12) | 1.4 (.93-2.1) | 96 (9.2) |
| CT/TT | 526 (55) | 1.0 (.84-1.2) | 144 (53) | 0.94 (.72-1.2) | 106 (53) | 0.93 (.69-1.3) | 86 (57) | 1.1 (.77-1.5) | 190 (57) | 1.1 (.85-1.4) | 573 (55) |
| *p for trend* |  | *0.30* |  | *0.94* |  | *0.63* |  | *0.28* |  | *0.20* |  |
| **rs2813545** |  |  |  |  |  |  |  |  |  |  |  |
| AA | 621 (65) | 1.0 | 171 (63) | 1.0 | 137 (68) | 1.0 | 102 (68) | 1.0 | 209 (63) | 1.0 | 662 (63) |
| AG | 287 (30) | 0.89 (.74-1.1) | 85 (31) | 0.96 (.72-1.3) | 53 (26) | 0.75 (.53-1.1) | 39 (26) | 0.74 (.50-1.1) | 110 (33) | 1.0 (.78-1.3) | 344 (33) |
| GG | 48 (5.0) | 1.3 (.82-2.0) | 14 (5) | 1.3 (.71-2.5) | 11 (5.5) | 1.3 (.63-2.5) | 10 (6.6) | 1.8 (.85-3.7) | 13 (3.9) | 1.1 (.55-2.0) | 41 (3.9) |
| AG/GG | 335 (35) | 0.93 (.78-1.1) | 99 (37) | 1.0 (.76-1.3) | 64 (32) | 0.80 (.58-1.1) | 49 (32) | 0.84 (.58-1.2) | 123 (37) | 1.0 (.79-1.3) | 385 (37) |
| *p for trend* |  | *0.86* |  | *0.73* |  | *0.43* |  | *0.86* |  | *0.85* |  |
| **rs910416** |  |  |  |  |  |  |  |  |  |  |  |
| TT | 272 (28) | 1.0 | 80 (30) | 1.0 | 58 (29) | 1.0 | 46 (30) | 1.0 | 88 (26) | 1.0 | 291 (28) |
| CT | 472 (49) | 1.0 (.81-1.2) | 131 (48) | 0.94 (.69-1.3) | 94 (47) | 0.92 (.64-1.3) | 76 (50) | 0.95 (.64-1.4) | 170 (51) | 1.1 (.83-1.5) | 511 (49) |
| CC | 215 (22) | 0.95 (.74-1.2) | 60 (22) | 0.90 (.62-1.3) | 49 (24) | 1.0 (.67-1.5) | 29 (19) | 0.77 (.47-1.3) | 76 (23) | 1.0 (.73-1.5) | 244 (23) |
| CT/CC | 687 (72) | 0.98 (.81-1.2) | 191 (70) | 0.93 (.69-1.2) | 143 (71) | 0.95 (.68-1.3) | 105 (70) | 0.89 (.61-1.3) | 246 (74) | 1.1 (.83-1.4) | 755 (72) |
| *p for trend* |  | *0.69* |  | *0.56* |  | *0.99* |  | *0.32* |  | *0.81* |  |
