# Supplementary material for: Polymorphisms in the Estrogen Receptor 1 and Vitamin C and Matrix Metalloproteinase Gene Families Are Associated with Susceptibility to Lymphoma
Source: PLoS One. 2008 Jul 30;3(7):e2816. doi: 10.1371/journal.pone.0002816 (PMC2474696; doi:10.1371/journal.pone.0002816)
Supplement: Table S5 — A priori hypotheses tested. (0.06 MB DOC) [file pone.0002816.s009.doc]

**Table S5*. A priori*** hypotheses tested.

| **Pathway/Gene Function** | |
| --- | --- |
| Steroidogenesis | We recently identified NHL risk alleles in *CYP17A1[7,10]* and catechol-O-methyltransferase (*COMT*), key genes involved in estradiol production and metabolism. We also found that SNPs in the prolactin (*PRL*) gene, which influences lymphocyte prolactin levels, were associated with lymphoma risk [7]. These results highlight the potential relevance of variation in genes involved in sex hormone synthesis/metabolism as immune modulators that affect apoptosis, activation and proliferation of immune cells that may promote lymphomagenesis. |
| Immunity | In a large consortial genetic study of NHL, we previously identified SNPs in *TNF* and *IL10* genes associated with high TNF-α and low IL-10 producer phenotypes as NHL risk alleles [59]. TNF-α is a pro-inflammatory cytokine that exerts anti-apoptotic effects on B-cells, while IL-10 hinders the inflammatory response by inducing apoptosis in mast cells and macrophages. In pooled analyses of two large case-control studies, we also found associations with NHL and variants in the caspase activating recruitment domain 15 (*CARD15*) and toll-like receptor 4 (*TLR4*) genes [4] that encode pro-inflammatory mediators integral as a first line of defense against viral and bacterial infection. Other genes such as the leukocyte adhesion molecules, vascular cell adhesion molecule-1 (*VCAM1*) and intercellular adhesion molecule-1 (*ICAM1*) of the immunoglobulin superfamily, also play essential roles in innate immune function. Because they are important in developing a persistent antigenic response, they may play a key role in NHL risk. |
| Cell signaling/apoptosis | Homeostasis of the immune system is regulated by integrated signaling processes, which also may influence the risk of lymphoma. A deregulated apoptotic mechanism involving t(14;18) and bcl2 over-expression is usually observed in lymphomas, particularly in indolent FL. Persistent NFκB activity may play a key role in growth deregulation and anti-apoptotic behavior, factors likely relevant in the pathogenesis of lymphoma. The study of *BCL2*, NFκB genes such as *NFKBIB* and others involved in cell death and signaling pathways may help to clarify whether associations exist between SNPs in these genes and lymphoid neoplasms. |
| Sunlight/vitamin D | Recent studies suggest an inverse relationship between exposure to sunlight and NHL risk [60,61]. Ecologic and case-control studies also provide evidence of inverse associations between ambient UV exposure and NHL incidence and mortality. We hypothesized that investigations of SNPs in the vitamin D receptor and in pigmentation genes may provide further clues of the importance of the vitamin D pathway as a mediator of the potentially inhibitory effects of UVexposure on lymphomagenesis. |
| Xenobiotic metabolism/  oxidative stress | Reactive oxygen species are implicated in inflammatory conditions and are likely involved in the pathogenesis of lymphoma. In a recent pooled analysis of 1,593 NHL cases and 2,517 controls, we found that SNPs in the superoxide dismutase 2 (*SOD2*) and glutathione peroxidase 1 (*GPX1*) genes influenced lymphoma risk [5]. Conversion of the superoxide anion to hydrogen peroxide is catalyzed by SOD2, while GPX is an important antioxidant enzyme that catalyzes detoxification of hydrogen peroxide. Thus, pro-oxidant mechanisms that enhance free radical damage may be very relevant in lymphomagenesis, particularly where chronic inflammation may increase risk of neoplastic changes. |
| Energy balance | We and others have found associations between obesity and hemato-lymphopoietic cancers including NHL, leukemia and multiple myeloma [62]. Obesity is associated with impaired immune function and generalized inflammation characterized by increased circulation of pro-inflammatory mediators such as leptin, IL-6 and C-reactive protein. We recently identified links between NHL and polymorphisms in the leptin (*LEP*), leptin receptor (*LEPR*), neuropeptide Y (*NPY*) and ghrelin (*GHRL*) genes [9,11] that are key genes in body weight homeostasis. Thus, from both epidemiological and genetic data, there is evidence to suggest that obesity may be a causal factor in the pathogenesis of N HL through its adverse action on immune system functions. |
| Cholesterol synthesis/  metabolism | We have previously shown a protective effect with cholesterol lowering drug use in risk of NHL[63,64] that has now been replicated in a large NHL study[65]. Certain statins block adhesion molecule interactions, important for cell-cell communication including those between EBV-transformed B cells. These same statins inhibit NF-kappaB activation and induce apoptosis of transformed B cells[66]. Cholesterol depletion also leads to increased apoptosis of breast, prostate [67] and other epithelial cancer cell types. Thus, SNPs in cholesterol synthesis, metabolism and transport genes may influence the bioavailability of cholesterol, which may ultimately alter risk of NHL. |
| Diet  Folate metabolism  Vitamin C uptake | Fruit and vegetable intake has been inversely associated with NHL [14,15], which may partly relate to the relatively high content of vitamin C and folate found in these foods. As a scavenger of reactive oxygen species, vitamin C may block DNA damage induced by oxidative stress by quenching free radical formation. Vitamin C also plays an anti-tumorigenic role in supporting proper collagen formation and matrix stabilization [22]. Folate deficiency or genetic variation in folate metabolic pathways can influence DNA methylation patterns and impede DNA synthesis and repair mechanisms. Epigenetic silencing of tumor suppressor and B-cell specific genes is an important mechanism in hematopoietic malignancies. Gene variants that influence methylation processes may promote lymphoma through modulation of the rate of chromosomal translocations, efficiency of DNA repair and DNA methylation status [2]. |
